# Ontogeny and Homology of the Shoulder Joint and Forelimb Muscles in Frogs and Toads (Lissamphibia: Anura)

**DOI:** 10.1002/jmor.70158

**Published:** 2026-08-19

**Authors:** Robin Saulnier Masson, Karolin Engelkes

**Affiliations:** ^1^ Leibniz Institute for the Analysis of Biodiversity Change Hamburg Germany; ^2^ University of Hamburg Hamburg Germany

**Keywords:** Anura, frog, homology, metamorphosis, muscle anatomy, ontogeny

## Abstract

Recent studies revealed inaccuracies in the naming and homologization of anuran shoulder joint muscles, and highlighted the value of muscle ontogeny and innervation to derive homology hypotheses. To expand the taxonomic range of previous work and add forearm muscles, the ontogenetic development, innervation, and morphology of shoulder joint and forelimb muscles were described based on tadpoles (Gosner Stages 31–41; Nieuwkoop‐Faber Stages 53–54 to 59) and adult specimens of 11 anuran species. Digital dissections of volume data (histology, episcopic microtomy, µCT) were performed and 3D reconstructions generated. The developmental and anatomical observations were used to support existing or suggest new hypotheses on muscle homology across species. Results indicate that, like in all tetrapods, the shoulder joint and forelimb muscles are ontogenetically derived from the dorsal and ventral pre‐muscle masses in the developing limb, which split and differentiate into individual muscles. The splitting pattern appears mostly conserved across sampled species. Interspecific variations are observed in the shoulder joint muscles, which comprise homologous muscles being present as one muscle unit in some species and subdivided into two heads or portions in others. The *m. supracoracoideus*, for example, is undivided in some species but splits into *portiones anterior* and *posterior* during ontogeny in others, resulting in a muscle with a common insertion but two distinct origins. Interspecific variation among forearm muscle units concerns morphological differences rather than divergence in developmental splitting pattern, the most notable examples being the *m. extensor digitorum communis longus* and the *m. abductor indicis longus*. For the former, we observed three distinct heads in some species and suggest naming them *capita digiti medii, annularis*, and *minimi*, respectively, for digits III–V. To reflect homology across Anura, we recommend using the name *m. abductor indicis longus* instead of *m. supinator manus* as previously used in *Ascaphus truei*.

## Graphical abstract

The ontogenetic development, innervation, and morphology of the shoulder joint and forelimb muscles were investigated in 11 anuran species. The results reveal largely conserved splitting patterns of the dorsal and ventral pre‐muscle masses, alongside interspecific variation in muscle subdivision and morphology. These data provide new hypotheses on the homology of shoulder joint and forelimb muscles and support a revised muscle nomenclature for Anura.

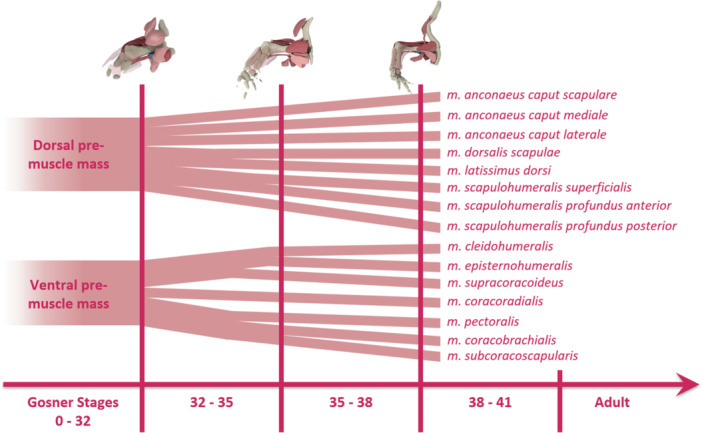

## 1 Introduction

The evolution of the muskuloskeletal systems of the fish pectoral fins, tetrapod forelimbs, and their pectoral girdles has long been studied to establish morphological and anatomical homologies across vertebrates and to understand the evolutionary transformations underlying the fins‐to‐limbs transition (e.g., Romer 1924; Diogo et al. 2016; Molnar et al. 2018). While the evolution and resulting homologies of skeletal structures in the pectoral girdle and forelimbs are well‐documented and supported by the fossil record (e.g., McGonnell 2001; Coates et al. 2002; Shubin et al. 2006; Coates et al. 2008; Shubin et al. 2009; Ahlberg 2011; Ponomartsev et al. 2017), the evolution of the related soft tissues remains largely unknown due to its poor preservation during fossilization and insufficient evidence to support hypotheses on soft tissue evolution (Soliz et al. 2018; Molnar et al. 2018). However, recent studies have attempted to establish forelimb muscle homologies across tetrapods (Diogo and Abdala 2007; Abdala and Diogo 2010; Smith‐Paredes et al. 2022), and reconstruct the evolutionary transitions of muscles from fins to limbs (Diogo et al. 2016; Molnar et al. 2018).

Despite the remarkable species diversity of extant amphibians, studies on the evolution of pectoral girdle and forelimb anatomy have predominantly focused on caudate species (Romer 1922; Francis 1934; Diogo and Abdala 2007; Diogo et al. 2016; Molnar et al. 2018; but see, e.g., Abdala and Diogo 2010 and Engelkes et al. 2021 for the consideration of anuran species). This approach overlooks the fact that the independent evolutionary histories of the Caudata are as long as that of its sister taxa, the Anura (e.g., Jetz and Pyron 2018). Extant caudate species therefore likely also show evolutionarily derived morphological traits if compared to the last common ancestor of the Caudata and Anura. Thus, including the Anura into studies of pectoral girdle and forelimb evolution would allow for a better‐informed assessment of the muscle character states in the last common ancestor of the Anura and Caudata and, thereby, contribute to a more reliably reconstructon of the morphological changes accompanying the fins‐to‐limb transition.

Previous studies on the shoulder joint and forelimbs identified and named all the soft tissue units in various anuran species (e.g., Gaupp 1896; Grobbelaar 1924; Bigalke 1927; Ritland 1955; Abdala and Diogo 2010; Porro and Richards 2017; Engelkes et al. 2021). Nonetheless, certain inconsistencies exist regarding the identification and homologization of muscle units, as well as the nomenclature employed for the muscles (Engelkes et al. 2021). Concerning the pectoral girdle, for example, Grobbelaar (1924; *Xenopus laevis*) synonymized his *m. mylo‐pectori‐humeralis* with Gaupp (1896; *Rana* spp.) *m. deltoideus pars episternalis*, while Ritland (1955; *Ascaphus truei*) termed this muscle *m. deltoideus [d1, episternohumeralis]*, thereby implying the homology of the corresponding muscle units. Further, using Grobbelaar (1924) muscle descriptions, Porro and Richards (2017; *Xenopus laevis*) synonymize the muscle they refer to as the proximal portion of *m. sternoradialis* with the *m. coraco‐radialis* of Grobbelaar; examining the illustrations and texts of both studies rather suggests that the proximal portion of *m. sternoradialis* in Porro and Richards actually corresponds to Grobbelaar's *m. mylo‐pectori‐humeralis*, while his *m. coraco‐radialis* corresponds in fact to Porro and Richards's *m. coracohumeralis*. Such inconsistencies may lead to misunderstandings of the anuran pectoral girdle anatomy and contradict the homology hypotheses proposed by Grobbelaar (1924) and Ritland (1955), which are also supported by Engelkes et al. (2021). Whereas studies of the musculoskeletal system of the pectoral girdle are underrepresented, studies of the forearm are even less common, likely due to the challenges in distinguishing the muscle units given their large number and small size (e.g., Porro and Richards 2017). Moreover, some studies attempt to establish a nomenclature that takes into account the evolution and homologies of the limb muscles across all major tetrapod groups (Abdala and Diogo 2010; Diogo and Ziermann 2014), challenging the assessment of homologies within anurans. For example, the *m. brachioradialis* and the *m. supinator* are forearm muscles shared by several tetrapods' groups (Abdala and Diogo 2010; Smith‐Paredes et al. 2022) but lacking a homologous muscle in the majority of anatomical descriptions of the forearm of anurans. Diogo and Ziermann (2014) introduced these muscle names for *Eleutherodactylus coqui* by grouping several distinct muscles, like the *m. flexor antibrachii lateralis superficialis* and the *m. flexor antibrachii lateralis profundus* (*sensu* Duellman and Trueb 1986) and terming them *m. brachioradialis,* whereas they renamed the *m. flexor antibrachii lateralis profundus* as *m. supinator*. In doing so, Diogo and Zierman imply homologies of these muscles across tetrapode groups without additional support, for example, from ontogeny, innervation, muscle evolution within tetrapodes, or the fossil record. Additionally, both intraspecific and interspecific morphological variation can impose challenges with regard to the homologization of muscles units across anuran species. For instance, sexual dimorphism in forearm muscles has been documented in several anuran species (Gaupp 1896; Lee Clark and Peters 2006; Navas and James 2007). Interspecific differences in muscle morphology are illustrated by the case of the *m. flexor antibrachii medialis*: Gaupp (1896) and Bigalke (1927) reported this muscle in species of the genus *Rana* and in *Bufo bufo*, whereas Ritland (1955) considered it part of the *m. flexor carpi radialis* in *Ascaphus truei*.

While the inconsistencies mentioned above create challenges in identifying homologous muscles of the shoulder joint and forelimbs across anuran species, Engelkes et al. (2021) address this issue by suggesting interspecific homologies for the muscles spanning the shoulder joint for a few, rather distantly related species. However, the sparse phylogenetic sample in the previous work and the complete lack of forearm muscle homologization remain an obstruction to a reconstruction of the muscle morphology in the last common ancestor of the Anura. This ancestral state reconstruction, however, would help to clarify muscle homologies between the Anura and the Caudata, contributing to better integration of these two taxa into the broader context of pectoral girdle and forelimb evolution.

In this study, we analyzed the muscles of the pectoral girdle and forearm of anurans in order to suggest well‐supported hypotheses on interspecific homologies. Expanding the work of Engelkes et al. (2021), the innervation and ontogenetic development of the shoulder joint and forearm muscles were assessed and described in seven species distributed across the anuran phylogeny to support these homology hypotheses. The ontogenetic taxonomic sample represents two ancient lineages [Archaeobatrachia: Bombinatoridae: *Bombina orientalis* (Boulenger 1890); Mesobatrachia: Pipidae: *Xenopus laevis* (Daubin 1802), *Xenopus tropicalis* (Gray 1864)] and the two major neobatrachian groups [Hyloidae: Bufonidae: *Rhinella marina* (Linnaeus 1758); Hylidae: *Trachycephalus resinifictrix* (Goeldi 1907); Ranoidea: Microhylidae: *Kaloula pulchra* (Gray 1831); Ranidae: *Rana temporaria* (Linnaeus 1758)]. In addition, specimens from late metamorphic stages and adults of *Alytes obstetricans* (Laurenti 1768) [Archaeobatrachia: Alytidae], *Ascaphus truei* (Stejneger 1899) [Archaeobatrachia: Ascaphidae], *Bufo bufo* (Linnaeus 1758) [Neobatrachia: Hyloidae: Bufonidae] and *Limnodynastes peronii* (Duméril and Bibron 1841) [Neobatrachia: Myobatrachoidea: Limnodynastidae] were used to complement the description of the various muscles, as well as to expand the phylogenetic range. Furthermore, existing descriptions of the forearm muscles in some species [various species of *Rana* in Gaupp 1896; *Xenopus laevis* in Grobbelaar 1924; *B. bufo* in Bigalke 1927; *A. truei* in Ritland 1955] have been reassessed to identify inconsistencies in muscle naming and to propose a consistent nomenclature.

## 2 Materials and Methods

### 2.1 Specimens and Usages

A total of 34 specimens of 11 species (Supporting Information S1: Material S1), larvae ranging from Gosner Stages (GS) 31–41 (Gosner 1960), and Nieuwkoop‐Faber (NF) Stages 53–54 to 59 (Nieuwkoop and Faber 1994), were analyzed to investigate the ontogenetic development and innervation of the shoulder joint and forearm muscles using histological serial sectioning and three‐dimensional (3D) reconstruction. Regarding the ontogenetic development of Pipidae, larval stages of the model organisms *Xenopus laevis* and *X. tropicalis* were examined to provide an anatomical overview of the genus *Xenopus* (Wagler 1827). The latest larval stages and additionally adult specimens were analyzed to reassess previously published anatomical descriptions (Gaupp 1896; Grobbelaar 1924; Bigalke 1927; Ritland 1955) of those or closely related (according to the phylogeny by Alexander Pyron and Wiens 2011) species. As no histological serial sections were available for *Bufo bufo*, this species was not considered with regard to muscle innervation. In the same way, as no ontogenetic series were available for *Limnodynastes peronii*, this species was only used for the description of musculature and innervation, and illustration. Specimens sectioned for this study have been deposited in the collection of the Museum of Nature Hamburg (formerly Zoologisches Museum Hamburg, ZMH). No specimens were euthanized particularly for this study.

### 2.2 Histological Serial Sectioning and Digitization

Larval specimens selected for histological serial sectioning were decalcified and embedded in Paraplast Plus (Leica Biosystems). The resulting blocks were sectioned using a rotatory microtome (Microm HM 340 E; Microm International GmbH) with slice thicknesses between 6 and 10 µm depending on the size of the specimens. The sections were mounted on glass slides and stained following a modified Azan staining protocol (adapted from Zbären 1966), which included 3 min of staining in Kernechtrubin (Morphisto GmbH), 7 min of differentiation in 5% phosphotungstic acid (Carl Roth GmbH + Co. KG), 7–9 min of staining in aniline blue‐orange G (Morphisto GmbH), and 5 min of differentiation in 96% ethanol.

Histological serial sections of the pectoral girdle and forelimb regions were digitized with a slide scanner (Olympus SlideView VS200; Olympus Corporation) and processed in Fiji (based on ImageJ version 2.9.0; Schindelin et al. 2012; Schneider et al. 2012) for brightness, contrast, sharpness, and canvas size adjustments. The digital image stacks were first aligned with a rigid alignment, followed by an elastic alignment using the Fiji plugin TrakEM2 (Cardona et al. 2012). The resulting aligned volumes were then converted into 16‐bit grayscale images for use in the software Amira V6.0.1 (Thermo Fisher Scientific).

### 2.3 Episcopic Microtomy

The episcopic image datasets of *Kaloula pulchra* (ZMH A15064) and *Xenopus laevis* (ZMH A05087) were obtained following the methodology described in detail by Engelkes et al. (2018). Briefly, (sub‐)adult specimens selected for episcopic microtomy were decalcified and lead‐stained, followed by embedding with Paraplast Plus. Fiducial points were introduced into the block to improve image alignment accuracy during subsequent processing steps. Serial sections of 10 µm thickness were cut from the block surface using a rotary microtome (Microm HM 340 E; Microm International GmbH). Every 30 µm for *X. laevis* and 40 µm for *K. pulchra*, an image of the block surface was captured (camera: Nikon D7200; macro lens: Nikon AF‐S VR Micro‐Nikkor 105 mm 1:2, 8G IF‐ED; Nikon Corporation). The block surface was then stained with a 7% sodium sulfide solution, and a second image was acquired. Images were digitally processed using IrfanView (Irfan Skiljan, http://www.irfanview.com; NEF or RAW to TIF conversion) and Fiji (subtraction of corresponding unstained and stained images, followed by brightness and contrast adjustment). One to two fiducial points at each corner were used to align the image stack in Amira. The resulting volume was subsequently converted to 16‐bit grayscale in Fiji. At defined distance intervals, histological sections were mounted on glass slides, stained using a modified Azan staining protocol, and digitized as above. The digitized sections were then rigidly aligned in Amira. *Alytes obstetricans* (ZMH A12442) was used from Engelkes et al. (2021).

### 2.4 MicroCT Scanning

Specimens of *Bufo bufo* (ZMH A04660), *Rana temporaria* (ZMH A11310), and *Ascaphus truei* (CAS 252678) were, respectively, stained in 1% Lugol's solution, 1% iodine in 70% ethanol (EtOH), and 2% Lead(II)‐Acetat‐Trihydrat in increasing EtOH concentrations from 80% to 96% for 6–7 days with the staining solution being changed every few days (protocols modified from Metscher 2009 and Pauwels et al. 2013).

The specimens of *B. bufo* (ZMH A04660) and *R. temporaria* (ZMH A11310) were scanned using a YXLON FF20 CT (YXLON International GmbH). The specimen of *A. truei* (CAS 252678) was scanned using a Skyscan 1172 (Bruker Corporation). Volumes were reconstructed from X‐ray projections using the software delivered with the respective scanner.

### 2.5 Segmentation, 3D Reconstruction, and Visualization

All volumes were imported into Amira. The shoulder joint muscles, forearm muscles, and relevant skeletal elements (in larvae: only chondrified or ossified parts; condensations of mesenchymal cells or perichondrium/peristeum were not considered) were digitally dissected (i.e., segmented) using *Segmentation Editor* with *Brush* or *Magic Wand* tools. Bone and cartilage were not distinguished in all specimens. Due to tissue visibility, tissue damage, or the orientation of the forearm during sectioning (we opted for cross‐sections as longitudinal sections result in a significant loss of quality and precision), the segmented side is not the same for all specimens. Consequently, the GS 35 of *Rhinella marina* larva (ZMH A14931) was mirrored to achieve consistency with the two other specimens in the illustrations of the *R. marina* ontogeny. Additionally, the nerves innervating the shoulder joint and forearm muscles were segmented in the *L. peronii* larva (ZMH A14889), and in the adult specimen of *X. laevis* (ZMH A05087). The aligned digitized histological sections in original colors were used for comparison if possible and necessary during segmentation. If segmentations existed from previous work, those were checked, corrected if necessary, and complemented by segmenting the structures relevant for the present study.

For specimens selected for illustrations, polygon surfaces of the segmented anatomical structures were generated and simplified by iterative reduction of polygon count and smoothing in Amira. Depending on the intended further use for illustrations, surfaces were directly visualized in Amira or exported in.obj format. The surface generation and export were accelerated using a custom macro (*MultiExport*; see Engelkes et al. (2018) for details). Surfaces of the *R. marina* specimens (ZMH A14928 and ZMH A14933) were reused from Engelkes et al. (2021) for shoulder joint muscles.

The simplified surfaces of the *R. marina* larvae, *L. peronii*, and *X. laevis* were imported into MODO V10.1v (The Foundry; https://www.foundry.com) and manually edited to correct artifacts, fill holes, and smooth the surfaces. Some nerves and small or thin muscles showed significant artifacts (i.e., discontinuity or holes), requiring major manual adjustments. While care was taken to maintain the important properties (i.e., relative position to other anatomical elements and connections between muscles and bones), the form and thickness of the surfaces may not fully reflect the actual conditions. The surfaces produced from *L. peronii* and *X. laevis* were used to illustrate all muscle units with their associated nerves and to highlight the range of interspecific anatomical variations observed in this study. All surfaces were given descriptive colors prior to image rendering. The images were arranged and labeled in Inkscape V1.3.2 (Inkscape: Open Source Scalable Vector Graphics Editor).

### 2.6 Muscle Homology Hypotheses and Nomenclature

We pragmatically define shoulder joint muscles as all muscles that span the shoulder joint. Similarly, muscles extending along the radioulna with at least half of their length or originating from the radioulna are defined as forearm muscles. These definitions are only used to delimit the anatomical scope of the study and don't constitute homology hypotheses; using these definitions resulted in all muscle units derived from the considered pre‐muscle masses being included in the descriptions such that there are no contradictions regarding homologizations across species. Hypotheses regarding the interspecific homology of muscle units (in terms of primary homology; de Pinna 1991) were proposed based on the criteria for homology established by Remane (1952). Muscle units were considered homologous if they exhibited a similar relationship to anatomical structures such as skeletal elements and joints (first criterion of Remane 1952) and could be linked through comparable intermediate stages during ontogenetic development (third criterion of Remane 1952; also see Kerr 1955). Therefore, muscle homology hypotheses were suggested by equally considering anatomical position and ontogenetic evidence. The homology of the skeletal elements across anurans is well established through osteological and paleontological studies (Duellman and Trueb 1986; Molnar et al. 2018), and they therefore provide independent anatomical references for assessing muscle homology. In addition, the delimitation of individual muscle units was informed by their developmental origin and subdivision patterns. Accordingly, one single, undivided muscle in one species was considered homologous to a subdivided muscle (i.e., two or more portions) in another species if all portions originated from the same developmental precursor and if that precursor was homologous to the (precursor of) the one musle in the other species. Innervation patterns in late larval stages or adults were also taken into account to support the suggested homologies, as innervation has proven useful for identifying and homologizing muscles (e.g., Romer 1924; Holliday and Witmer 2007). In both mammals and anurans, nerve axons enter the limb pre‐muscle masses almost concurrently with their formation (Harrison 1907; Hurren et al. 2015), supporting their consideration in ontogenetic studies. Moreover, the branching patterns of the main nerves supplying the forelimb muscles appear to be relatively conserved across tetrapods (Hirasawa and Kuratani 2018). However, it is important to consider that some studies (e.g., Cunningham 1890; Romer 1922; Haines 1935; Minkoff 1974) have challenged the reliability of nerve supply as a determinant of muscle homologies. In the light of these concerns, innervation was considered only as supporting evidence for homology hypotheses and was not used as an independent criterion for establishing homology. The position and relationship between muscle units and major nerves were documented across species to provide additional information on muscle organization. This approach has already been shown to be effective in establishing shoulder joint muscle homologies in Anura (Engelkes et al. 2021). We assumed that nerve supply could be misleading in cases where no common branching pattern was evident or where variations in minor nerves were present. Innervation and branching were assessed and compared between two specimens of the same species in *Kaloula pulchra, Limnodynastes peronii, Rana temporaria, Rhinella marina, Trachycephalus resinifictrix and X. tropicalis*. Only minor intraspecific differences were observed, supporting the use of innervation pattern as additional information to establish homology hypotheses.

Concerning the shoulder joint muscles (Table 1), we followed the nomenclature proposed by Engelkes et al. (2021), which integrates muscle homologies across six anuran species while preserving, as much as possible, the terminology established by Gaupp (1896), Bigalke (1927), or Ritland (1955). Similarly, we used the nomenclature and descriptions by Gaupp (1896) as base for the identification and naming of the forearm muscles as this nomenclature has been widely used in previouse studies (Grobbelaar 1924; Bigalke 1927; Jones 1933; Mahendra 1936; Ritland 1955; Burton 1983; Duellman and Trueb 1994; Burton 1996; Manzano et al. 2008; Baleeva 2009; Blotto et al. 2020). However, we used the term *m. flexor digitorum communis longus* for the muscle identified as *m. palmaris longus* by Gaupp (1896), following more recent interpretations of anuran forelimb muscle homologies (e.g., Howell 1935, 1936a, 1936b; Straus 1942; Abdala and Diogo 2010; Porro and Richards 2017; Blotto et al. 2020). To minimize confusion with the previous muscle terminology of the forearm, we have already applied the herein suggested muscle names in the results section, based on the evidence presented in this work. Whenever possible, we retained the muscle names established by Gaupp (1896) or Ritland (1955), particularly when their usage remained consistent in the literature and aligned with ontogenetic–and potentially evolutionary–patterns of muscle development. A comparative overview of the forearm muscle nomenclature across authors is provided in Table 2. For nerve terminology, we followed Gaupp (1899).

**Table 1:** Hypotheses on homologies of shoulder joint muscles across selected species and suggested muscle names, as well as implied synonyms among different authors. Dotted line: muscles not entirely separated (e.g., continuous at origin). Modified from Engelkes et al. (2021); *Xenopus laevis* newly included in this study.

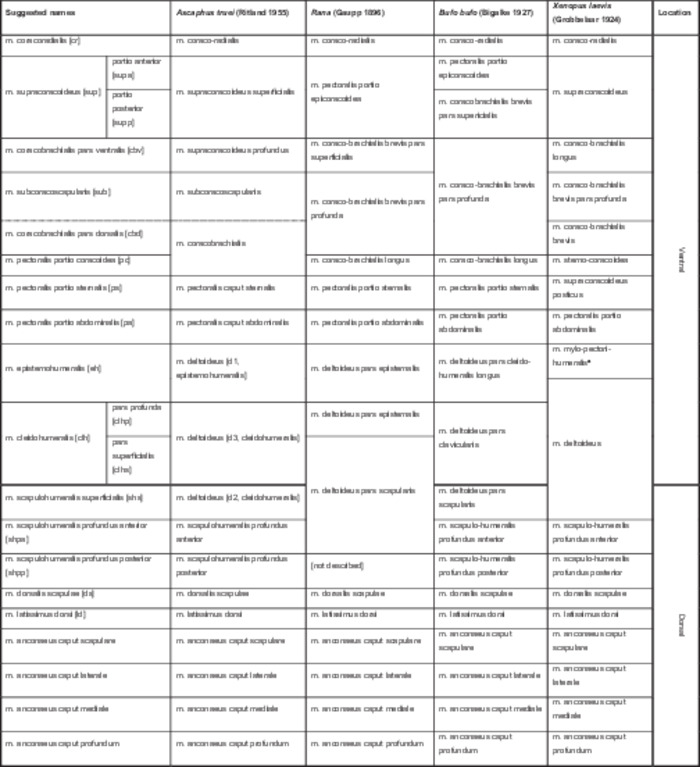

^a^ The m. episternohumeralis is considered to be two different muscles by Grobbelaar (1924), the m. mylo‐pectori‐humeralis and a part of the m. deltoideus.

**Table 2:** Hypotheses on homologies of forearm muscles across selected species and suggested muscle names, as well as implied synonyms of muscle names among different authors.

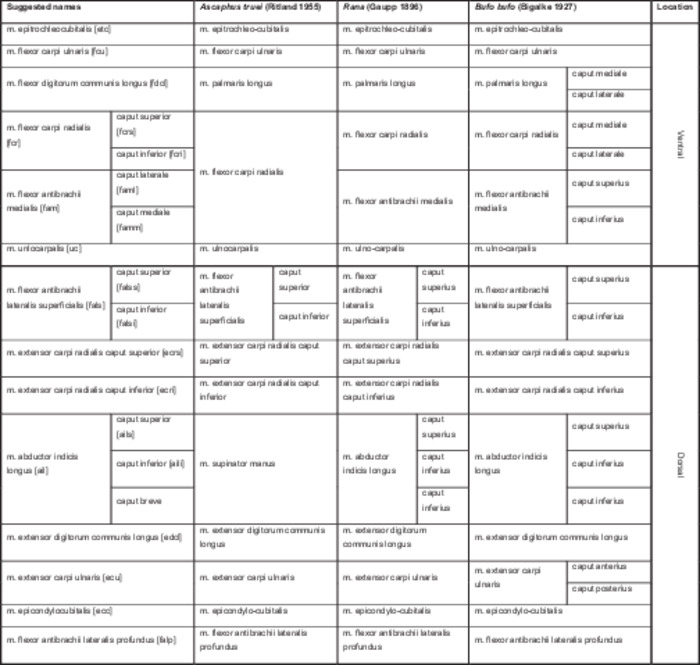

Abbreviations of muscles and nerves follow the standard Latin anatomical nomenclature convention (FIPAT 2019; Waschke et al. 2019), with *m*. and *mm*. denoting *musculus* (singular) and *musculi* (plural), respectively, *n*. denoting a nerve (*nervus)*, and *r*. denoting a branch of a nerve (*ramus*).

## 3 Results

Some observations regarding the musculature of the shoulder joint overlap with those reported by Engelkes et al. (2021), as several species are common to both studies. These observations are presented here following a re‐evaluation of the specimens and are repeated for the sake of completeness; these repetitions are not indicated below to keep the text concise and to avoid interrupting the flow of reading.

In all examined species, the development of the pectoral girdle and forelimb skeleton occurs in parallel with the associated musculature. In all species except for *Xenopus* spp., the skeleton shows advanced calcification and all shoulder joint and forelimb muscle units, although smaller than in the adults, are discernable before the forelimbs emerge from the branchial cavity. In *Xenopus* spp., the forelimbs emerge slightly earlier in development, with the skeleton still being mostly cartilaginous. Yet, all shoulder joint and forelimb muscle units present in the adult are already present at forelimb emergence. In all species, the skeletal elements develop from multiple centers of chondrification within pre‐cartilaginous clusters of mesenchymal cells. During ontogeny, these cartilaginous precursors grow and, in some regions, fuse to form the cartilaginous precursor of the skeleton prior to ossification.

**Figure 1:**
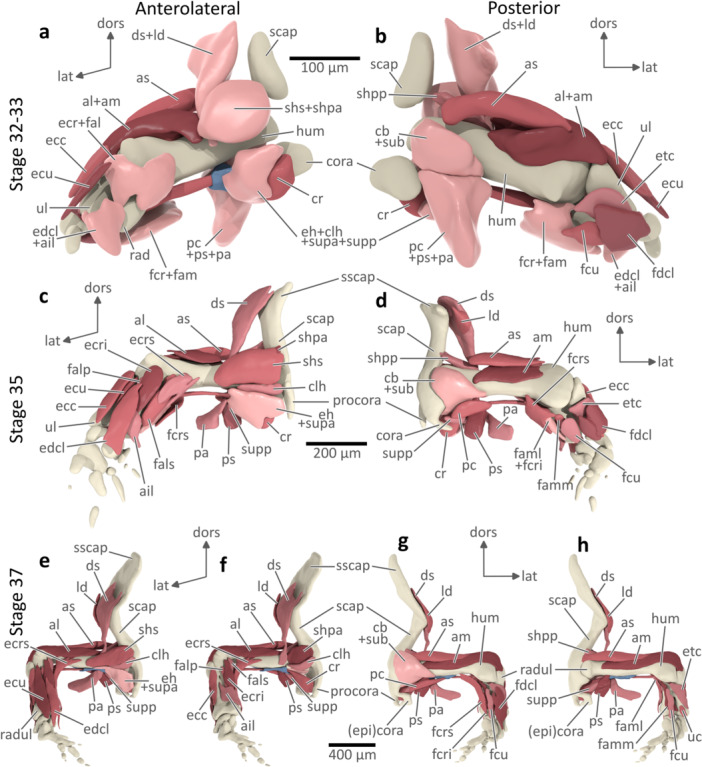
Ontogenetic development of the shoulder joint and forearm muscles in *Rhinella marina* (illustrated for right side). Anterolateral (left) and posterior (right) views. Surfaces derived from aligned histological serial sections. Red: muscles (different shades for better visual separation of adjacent muscles); beige: skeletal element with no distinction of bone and cartilage. (a, b) Gosner Stage 32–33 larva (ZMH A14928); separation of cb+sub and pc+ps+pa artificial based on differences in cell differentiation. (c, d) Gosner Stage 35 larva (ZMH 14931). (e–h) Gosner Stage 37 larva (ZMH A14933), muscle layers successively removed. ail: m. abductor indicis longus; al: m. anconaeus caput laterale; am: m. anconaeus caput mediale; as: m. anconaeus caput scapulare; cb: m. coracobrachialis; clh: m. cleidohumeralis; cora: coracoid; cr: m. coracoradialis; dors: dorsal; ds: m. dorsalis scapulae; ecc: m. epicondylocubitalis; ecri: m. extensor carpi radialis caput inferior; ecrs: m. extensor carpi radialis caput superior; ecu: m. extensor carpi ulnaris; edcl: m. extensor digitorum communis longus; eh: m. episternohumeralis; epicora: epicoracoid cartilage; etc: m. epitrochleocubitalis; fam: m. flexor antibrachii medialis; faml: m. flexor antibrachii medialis caput laterale; famm: m. flexor antibrachii medialis caput mediale; falp: m. flexor antibrachii lateralis profundus; fals: m. flexor antibrachii lateralis superficialis; fcr: m. flexor carpi radialis; fcri: m. flexor carpi radialis caput inferior; fcrs: m. flexor carpi radialis caput superior; fcu: m. flexor carpi ulnaris; hum: humerus; lat: lateral; ld: m. latissimus dorsi; pa: m. pectoralis portio abdominalis; pc: m. pectoralis portio coracoidea; fdcl: m. flexor digitorum communis longus; procora: procoracoid cartilage; ps: m. pectoralis portio sternalis; rad: radius; radul: radioulna; scap: scapula; shs: m. scapulohumeralis superficialis; shpa: m. scapulohumeralis profundus anterior; shpp: m. scapulohumeralis profundus posterior; sscap: suprascapula; sub: m. subcoracoscapularis; supa: m. supracoracoideus portio anterior; supp: m. supracoracoideus portio posterior; uc: m. ulnocarpalis; ul: ulna.

**Figure 2:**
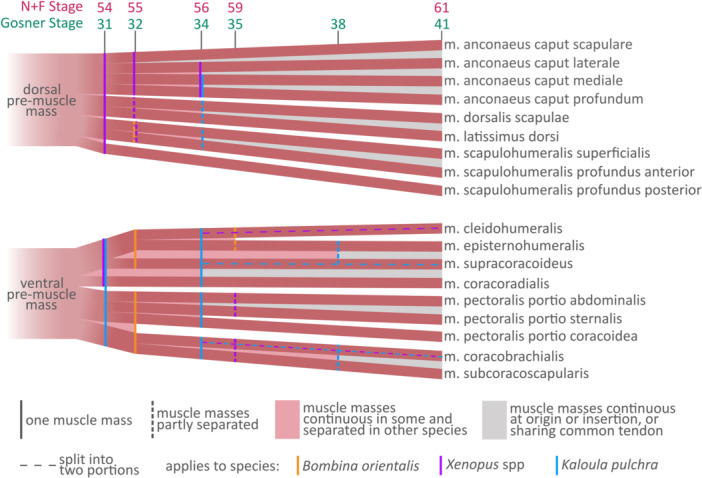
Generalized ontogenetic splitting pattern of the shoulder joint pre‐muscle masses located dorsal and ventral to the humerus. Timelines derived from larvae of *Bombina orientalis* (Gosner Stages 32, 35), *Kaloula pulchra* (Gosner Stages 31, 34, 38, 41), and *Xenopus* spp. (NF Stages 53–54, 55, 56, 59).

**Figure 3:**
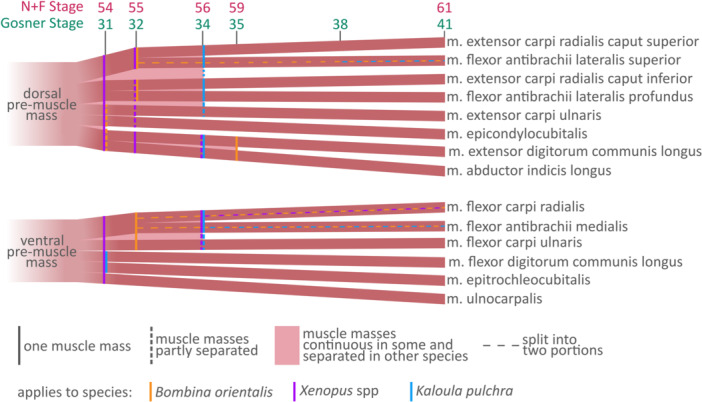
Generalized ontogenetic splitting pattern of the forearm pre‐muscle masses located dorsal and ventral to the radioulna. Timelines derived from larvae of *Bombina orientalis* (Gosner Stages 32, 35), *Kaloula pulchra* (Gosner Stages 31, 34, 38, 41), and *Xenopus* spp. (NF Stages 53–54, 55, 56, 59).

**Figure 4:**
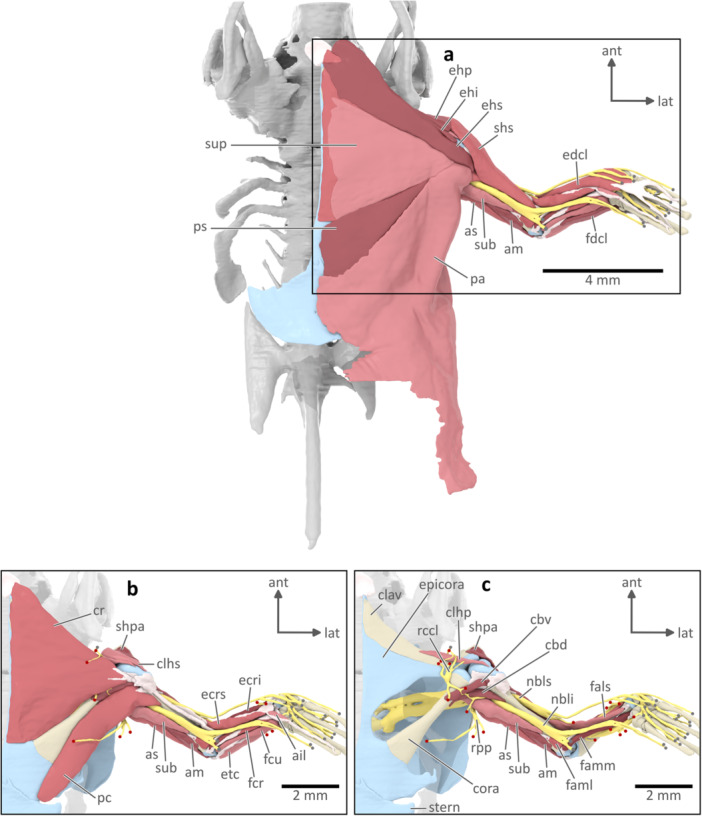
Shoulder joint and forearm muscles with respective nerve supplies in *Xenopus laevis* (ZMH A05087). Surfaces derived from aligned episcopic serial images. (a–c) Ventral views of the left pectoral girdle and forelimb, muscle layers successively removed. Spheres: nerve endings in muscle (red spheres) or cut (gray, no connection to shoulder joint or forearm muscles). Red: muscles (different shades for better visual separation of adjacent muscles); yellow: nerves; beige: bone; light blue: cartilage; light gray: skeletal element with no distinction of bone and cartilage; light pink: tendons. ail: m. abductor indicis longus; am: m. anconaeus caput mediale; as: m. anconaeus caput scapulare; ant: anterior; cbd: m. coracobrachialis pars dorsalis; cbv: m. coracobrachialis pars ventralis; clav: clavicula; clhp: m. cleidohumeralis pars profunda; clhs: m. cleidohumeralis pars superficialis: cora: coracoid; cr: m. coracoradialis; ecri: m. extensor carpi radialis caput inferior; ecrs: m. extensor carpi radialis caput superior; edcl: m. extensor digitorum communis longus; ehi: m. episternohumeralis portio intermedia; ehs: m. episternohumeralis portio superficialis; ehp: m. episternohumeralis portio profunda; epicora: epicoracoid cartiolage; etc: m. epitrochleocubitalis; fals: m. flexor antibrachii lateralis superficialis; faml: m. flexor antibrachii medialis caput laterale; famm: m. flexor antibrachii medialis caput mediale; fcr m. flexor carpi radialis; fcu: m. flexor carpi ulnaris; hum: humerus; lat: lateral; pa: m. pectoralis portio abdominalis; nbli: n. brachialis longus superior; nbls: n. brachialis longus superior; pc: m. pectoralis portio coracoidea; fdcl: m. flexor digitorum communis longus; ps: m. pectoralis portio sternalis; rccl: r. coraco‐clavicularis; rpp: r. pectoralis proprius; shpa: m. scapulohumeralis profundus anterior; shs: m. scapulohumeralis superficialis; stern: sternum; sub: m. subcoracoscapularis; sup: m. supracoracoideus.

A total of 21 muscle units are identified in the shoulder joint region and 19 in the forearm (Table 3). Those muscle units develop from a few pre‐muscle masses that split up and differentiate to form the individual muscles during ontogeny (Figures 1, 2, 3). However, a reduced number of muscle units is observed in certain species due to some muscles not splitting into distinct sub‐units as they do in other species (e.g., the *m. supracoracoideus*, which splits into two distinct portions in *Rhinella marina* (Figure 1), remains undivided in *Xenopus* spp. (Figure 4a).

**Table 3:** All muscles of the shoulder joint and forearm with their respective origins and insertions.

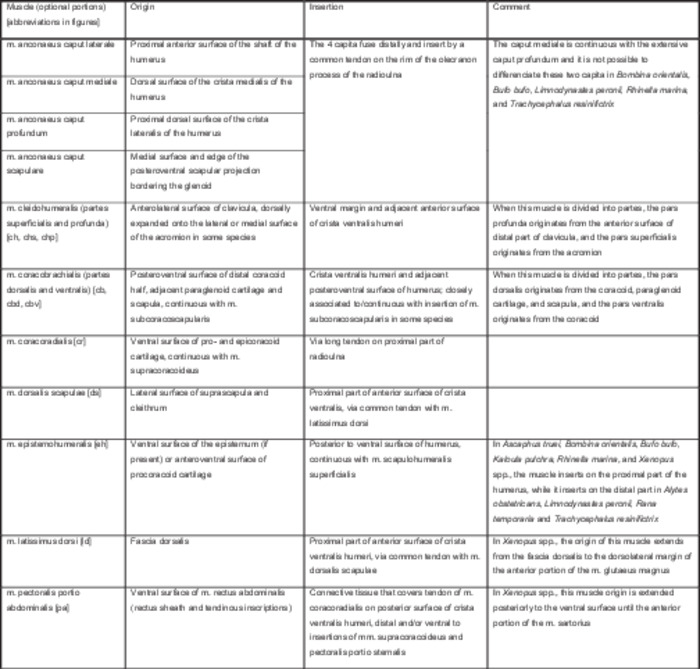
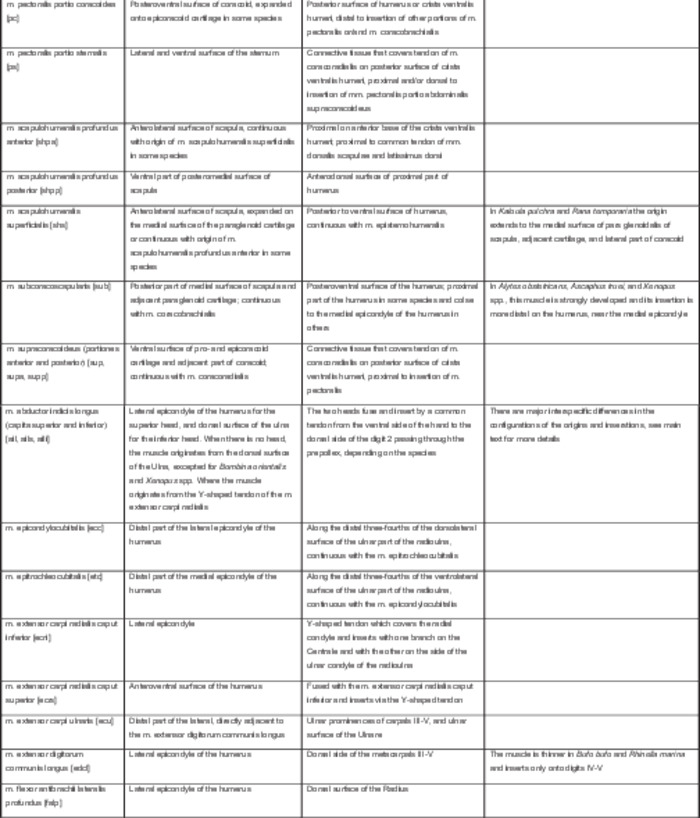
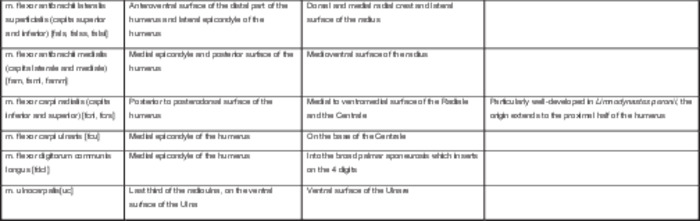

**Figure 5:**
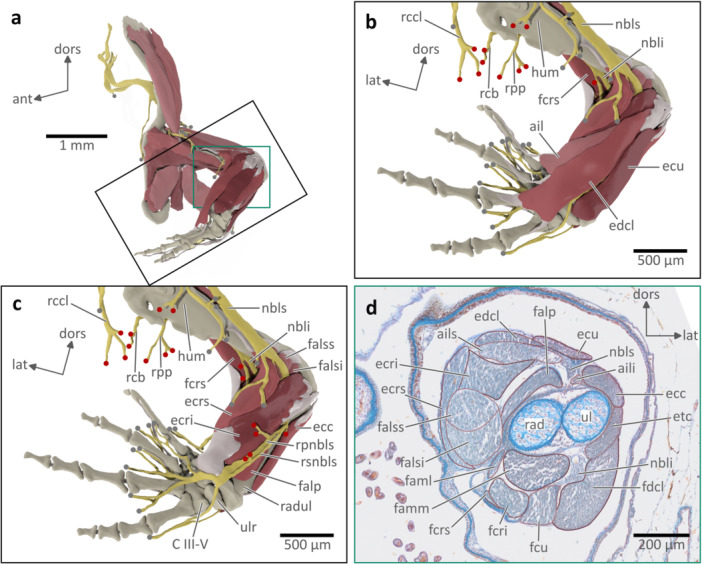
Forearm muscles with respective nerve supplies in *Limnodynastes peronii* (Gosner Stage 39, ZMH 14889). (a) Anterolateral view of left pectoral girdle and forelimb. (b, c) Anteromedial views of left forelimb. Surface derived from aligned histological serial sections, muscle layers successively removed. Spheres: nerve ending in muscle (red spheres) or cut (gray, no connection to shoulder joint muscles). Red: muscles (different shades for better visual separation of adjacent muscles); yellow: nerves; beige: skeletal element with no distinction of bone and cartilage. (d) Azan‐stained histological section of the proximal part of the forearm. Red lines: muscle delineation (different shades for better visual separation of adjacent muscles). ail: m. abductor indicis longus; aili: m. abductor indicis longus caput inferior; ails: m. abductor indicis longus caput superior; ant: anterior; C: carpal; dors: dorsal; ecc: m. epicondylocubitalis; ecri: m. extensor carpi radialis carpi inferior; ecrs: m. extensor carpi radialis caput superior; ecu: m. extensor carpi ulnaris; edcl: m. extensor digitorum communis longus; etc: m. epitrochleocubitalis; falsi: m. flexor antibrachii lateralis caput inferior; falss: m. flexor antibrachii lateralis caput superior; falp: m. flexor antibrachii lateralis profundus; famm: m. flexor antibrachii medialis caput mediale; faml: m. flexor antibrachii medialis caput laterale; fcri: m. flexor carpi radialis caput inferior; fcrs: m. flexor carpi radialis caput superior; fcu: m. flexor carpi ulnaris; hum: humerus; nbli: n. brachialis longus inferior; nbls: n. brachialis longus superior; fdcl: m. flexor digitorum communis longus; rad: radius; radul: radioulna; rccl: r. coraco‐clavicularis; rcb: r. coraco‐brachialis; rpnbls: r. profundus nervi brachialis longus superior; rpp: r. pectoralis proprius; rsnbls: r. superficialis nervi brachialis longus superior; ul: ulna; ulr: ulnare.

In all species examined, the myogenesis of the pectoral girdle and forelimb muscles begins with the formation of pre‐muscular cell clusters. These clusters surround the developing humerus and radioulna. During the early developmental stages considered herein (GS 32–33; NF 53–54), the clusters around the humerus give rise to an early *m. anconeus* and to three pre‐muscular masses (Figure 1a,b). The *m. anconeus* and one of these pre‐muscular masses are located approximately dorsal to the humerus, while the two others are located ventrally, with one being located anterior, the other posterior to the humerus. In parallel, on the ventral side of the forearm, two pre‐muscular masses are present and develop from a common ventral pre‐muscle mass: one on the proximal and radial part of the developing radioulna and the other on the ulnar part (Figure 1b; the ulnar pre‐muscle mass is already subdivided in the illustration). Dorsal to the developing radioulna, three muscle precursors arise from a common dorsal pre‐ muscule mass (Figure 1a; the ulnar pre‐muscle mass is already subdivided in the illustration). Among these three dorsal pre‐muscular masses, the most proximal one is located on the radial side and covers the *n. brachialis longus superior* proximal to its branching into *r. superficialis* and *r. profundus* (Figure 5c). The most distal pre‐muscular mass extends onto the dorsal surface of the hand, largely covering the *r. profundus*, while the middle one is located more ulnarly and superficial to the *r. superficialis* (Figure 5b,c). The identification of these pre‐muscular masses based on their relative position to and innervation by the rami of the *n. brachialis longus superior* is possible in most species, except for *Bombina orientalis* and *Xenopus* spp., in which the *r. superficialis* and *r. profundus* are absent. In *B. orientalis* and *Xenopus* spp., the relative position of the muscles and pre‐muscle masses within the forearm is sufficient for their identification. Nerves are present and in contact with all pre‐muscular masses and developing muscles in all examined stages.

With the progression of larval development, the single pre‐muscular mass located dorsal to the humerus and spanning the shoulder joint differentiates into the *mm. dorsalis scapulae*, *latissimus dorsi*, *scapulohumeralis superficialis*, *scapulohumeralis profundus anterior*, and *scapulohumeralis profundus posterior*. Similarly, the anteroventral pre‐muscular mass gives rise to the *mm. cleidohumeralis*, *episternohumeralis*, *supracoracoideus*, and *coracoradialis*, while the posteroventral mass divides into the *mm. pectoralis*, *coracobrachialis*, and *subcoracoscapularis* (Figure 1c–h). On the dorsal side of the forearm, the most distal pre‐muscular mass differentiates into the *mm. abductor indicis longus* and *extensor digitorum communis longus*, the ulnar mass into the *mm. extensor carpi ulnaris* and *epicondylocubitalis*, and the most proximal mass into the *mm. extensor carpi radialis caput superior* and *caput inferior*, *flexor antibrachii lateralis superficialis caput superior* and *inferior*, and *profundus* (Figure 1c,e,f). For this latter dorsal forearm mass, a consistent pattern of division is observed across all species: the mass first splits into two distinct muscle precursors, with the more radial and superficial one giving rise to the *mm. extensor carpi radialis caput superior* and *flexor antibrachii lateralis superficialis* (*capita superior* and *inferior*), and the more ulnar and deeper precursor developing into the *mm. extensor carpi radialis caput inferior* and *flexor antibrachii lateralis profundus*. On the ventral side of the forearm, the ulnar pre‐muscle mass splits into the *mm. epitrochleocubitalis*, *flexor digitorum communis longus* and *ulnocarpalis*, while the proximoradial pre‐muscular mass differentiates into the *mm. flexor carpi radialis caput superior* and *inferior*, *flexor antibrachii medialis caput laterale* and *caput mediale*, and *flexor carpi ulnaris* (Figure 1d,g,h).

### 3.1 Variation in Muscle Morphology Across Species

The 40 muscles are described in Table 3 with their origins and insertions; the following text only addresses interspecific differences. Mainly adult specimens are used in this comparison. However, if adult stages are missing, the latest larval stages (GS 41–43) examined in this study are considered, as all muscle units of adults are already present and differentiated in these stages.

**Figure 6:**
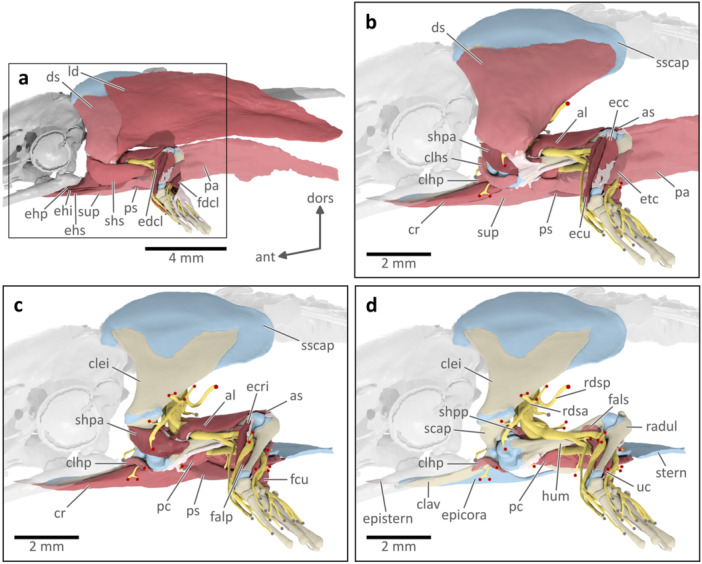
Shoulder joint and forearm muscles with respective nerve supplies in *Xenopus laevis* (ZMH A05087). Surfaces derived from aligned episcopic serial images. (a–d) Anterolateral views of the left pectoral girdle and forelimb, muscle layers successively removed. Spheres: nerve ending in muscle (red spheres) or cut (gray, no connection to shoulder joint or forearm muscles). Red: muscles/different shades for better visual separation of adjacent muscles; yellow: nerves; beige: bone; light blue: cartilage; light gray: skeletal element with no distinction of bone and cartilage; light pink: tendons. al: m. anconaeus caput laterale; as: m. anconaeus caput scapulare; ant: anterior; clav: clavicula; clei: cleithrum; clhp: m. cleidohumeralis pars profunda; clhs: m. cleidohumeralis pars superficialis; cora: coracoid; cr: m. coracoradialis; dors: dorsal; ds: m. dorsalis scapulae; ecc: m. epicondylocubitalis; ecri: m. extensor carpi radialis caput inferior; ecu: m. extensor carpi ulnaris; edcl: m. extensor digitorum communis longus; ehi: m. episternohumeralis portio intermedia; ehs: m. episternohumeralis portio superficialis; ehp: m. episternohumeralis portio profunda; epicora: epicoracoid cartilage; episterna: episternum; etc: m. epitrochleocubitalis; fals: m. flexor antibrachii lateralis superficialis; falp: m. flexor antibrachii lateralis profundus; fcu: m. flexor carpi ulnaris; hum: humerus; ld: m. latissimus dorsi; pa: m. pectoralis portio abdominalis; pc: m. pectoralis portio coracoidea; fdcl: m. flexor digitorum communis longus; ps: m. pectoralis portio sternalis; radul: radioulna; rdsa: r. dorsalis scapulae anterior; rdsp: r. dorsalis scapulae posterior; scap: scapula; shpa: m. scapulohumeralis profundus anterior; shpp: m. scapulohumeralis profundus posterior; shs: m. scapulohumeralis superficialis; sscap: suprascapula; stern: sternum; sup: m. supracoracoideus; uc: m. ulnocarpalis.

**Figure 7:**
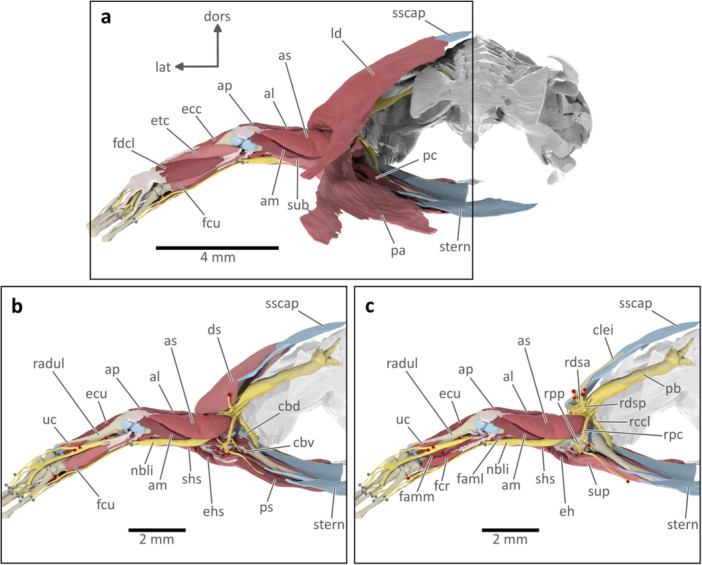
Shoulder joint and forearm muscles with respective nerve supplies in *Xenopus laevis* (ZMH A05087). Surfaces derived from aligned episcopic serial images. (a–c) Posterior views of the left pectoral girdle and forelimb, muscle layers successively removed. Spheres: nerve ending in muscle (red spheres) or cut (gray, no connection to shoulder joint or forearm muscles). Red: muscles/different shades for better visual separation of adjacent muscles); yellow: nerves; beige: bone; light blue: cartilage; light gray: skeletal element with no distinction of bone and cartilage; light pink: tendons. al: m. anconaeus caput laterale; am: m. anconaeus caput mediale; as: m. anconaeus caput scapulare; cbd: m. coracobrachialis pars dorsalis; cbv: m. coracobrachialis pars ventralis; clei: cleithrum; dors: dorsal; ds: m. dorsalis scapulae; ecc: m. epicondylocubitalis; ecu: m. extensor carpi ulnaris; ehs: m. episternohumeralis portio superficialis; etc: m. epitrochleocubitalis; faml: m. flexor antibrachii medialis caput laterale; famm: m. flexor antibrachii medialis caput mediale; fcr: m. flexor carpi radialis; fcu: m. flexor carpi ulnaris; hum: humerus; ld: m. latissimus dorsi; lat: lateral; nbli: n. brachialis longus inferior; pa: m. pectoralis portio abdominalis; pb: plexus brachialis; pc: m. pectoralis portio coracoidea; fdcl: m. flexor digitorum communis longus; ps: m. pectoralis portio sternalis; radul: radioulna; rccl: r. coraco‐clavicularis; rdsa: r. dorsalis scapulae anterior; rdsp: r. dorsalis scapulae posterior; rpc: r. pectoralis communis; rpp: r. pectoralis proprius; sub: m. subcoracoscapularis; sscap: suprascapular; stern: sternum; sup: m. supracoracoideus; uc: m. ulnocarpalis.

**Figure 8:**
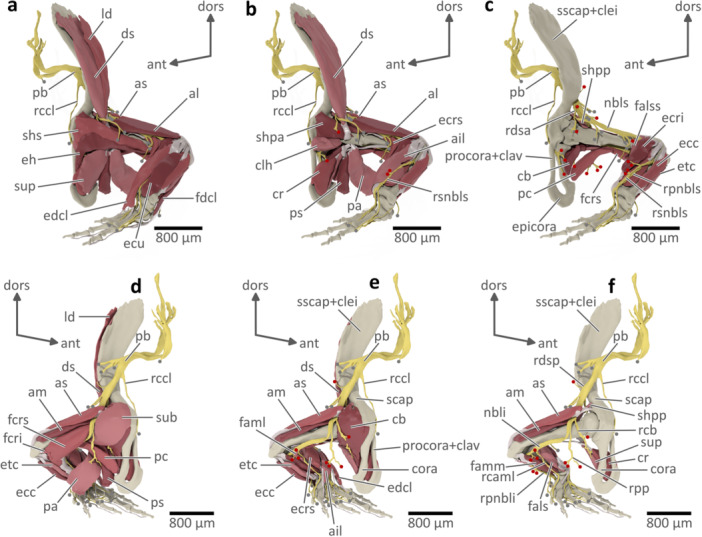
Shoulder joint and forearm muscles with respective nerve supplies in *Limnodynastes peronii* (Gosner Stage 39, ZMH A14889). Surfaces derived from aligned histological serial sections, muscle layers successively removed. Spheres: nerve ending in muscle (red spheres) or cut (gray, no connection to shoulder joint muscles). Red: muscles (different shades for better visual separation of adjacent muscles); yellow: nerves; beige: skeletal element with no distinction of bone and cartilage. (a–c) Anterolateral views of left pectoral girdle half. (d–f) Posteromedial views of left pectoral girdle half. ail: m. abductor indicis longus; al: m. anconaeus caput laterale; am: m. anconaeus caput mediale; as: m. anconaeus caput scapulare; ant: anterior; cb: m. coracobrachialis; clav: clavicula; clei: cleithrum; clh: m. cleidohumeralis; cr: m. coracoradialis; dors: dorsal; ds: m. dorsalis scapulae; ecc: m. epicondylocubitalis; ecri: m. extensor carpi radialis caput inferior; ecrs: m. extensor carpi radialis caput superior; ecu: m. extensor carpi ulnaris; edcl: m. extensor digitorum communis longus; eh: m. episternohumeralis; etc: m. epitrochleocubitalis; falp: m. flexor antibrachii lateralis profundus; falss: m. flexor antibrachii lateralis superficialis caput superior; faml: m. flexor antibrachii medialis caput laterale; famm: m. flexor antibrachii medialis caput mediale; fcri: m. flexor carpi radialis caput inferior; fcrs: m. flexor carpi radialis caput superior; fcu: m. flexor carpi ulnaris; ld: m. latissimus dorsi; nbli: n. brachialis longus inferior; nbls: n. brachialis longus superior; pa: m. pectoralis portio abdominalis; pn: plexus brachialis; pc: m. pectoralis portio coracoidea; fdcl: m. flexor digitorum communis longus; procora: procoracoid cartilage; ps: m. pectoralis portio sternalis; rcaml: r. cutaneus antibrachii et manus lateralis; rccl: r. coraco‐clavicularis; rcb: r. coraco‐brachialis; rdsa: r. dorsalis scapulae anterior; rdsp: r. dorsalis scapulae posterior; rpnbli: r. profundus nervi brachialis longus inferior; rpnbls: r. profundus nervi brachialis longus superior; rpp: r. pectoralis proprius; rsnbls: r. superficialis nervi brachialis longus superior; scap: scapula; shs: m. scapulohumeralis superficialis; shpa: m. scapulohumeralis profundus anterior; shpp: m. scapulohumeralis profundus posterior; sscap: suprascapula; sub: m. subcoracoscapularis; sup: m. supracoracoideus; uc: m. ulnocarpalis.

The *m. anconaeus* (Figures 1, 4, and 6, 7, 8) is divided into four *capita*: the *capita scapulare, mediale, laterale*, and *profundum*, in all species. In *Bombina orientalis, Bufo bufo, Limnodynastes peronii, Rhinella marina* and *Trachycephalus resinifictrix*, the *capita scapulare* and *laterale* are distinct, but the *capita mediale* and *profundum* are continuous and difficult to distinguish along their entire length.

The *m. latissimus dorsi* (Figures 1, 6a, 7a, and 8a,d) displays considerable variation across species. In *Alytes obstetricans*, *Ascaphus truei*, *Bombina orientalis*, and *Rana temporaria*, this muscle is located posterior and adjacent to the *m. dorsalis scapulae* along most of its length. In contrast, in *L. peronii* and *T. resinifictrix*, the anterior part of the *m. latissimus dorsi* extends anteriorly and superficially covers the posterior part or the entirety of the *m. dorsalis scapulae* (Figure 8a). The *m. latissimus dorsi* in *Bufo bufo*, *Kaloula pulchra*, and *Rhinella marina* is comparably thin, and it is only in contact with the *m. dorsalis scapulae* at its insertion (Figure 1e,f). In *Xenopus* spp., the origin of the *m. latissimus dorsi* extends from the *fascia dorsalis* near the spinous process of the second vertebra to the dorsolateral margin of the anterior portion of the *m. glutaeus magnus*. The ventral margin of the *m. latissimus dorsi* of *Xenopus* spp. and the dorsal margin of the *m. pectoralis portio abdominalis* are almost in contact (Figure 6a).

The *m. supracoracoideus* (Figures 1, 4a, 6b, 7c, 8a) is subdivided into a *portio anterior* and a *portio posterior* in *B. bufo, K. pulchra, R. marina* (Figure 1c–h) and *T. resinifictrix*, while it is not subdivided in *Ascaphus truei, Alytes obstetricans, Bombina orientalis, L. peronii, Rana temporaria*, and *Xenopus* ssp. (Figures 4a and 8a). In most species, this muscle occupies about the same area on the ventral side of the pectoral girdle independent of the muscle being subdivided or not. However, in *Rhinella marina* and *T. resinifictrix*, the two *portiones* are continuous at their origins but separate along their entire length, with the *portio anterior* being reduced in width. In *A. obstetricans* and *Ascaphus truei*, both *portiones* are of similar size. In *K. pulchra*, the *portio anterior* completely covers the *portio posterior*.

The *m. cleidohumeralis* (Figures 1, 4b,c, 6b–d, and 8b) is composed of two muscle units, the *pars profunda* and *pars superficialis*, in *Rana temporaria*, *T. resinifictrix*, and *Xenopus* spp. (Figures 4b,c and 6b–d), whereas it consists of a single muscle unit in the other species examined herein (Figures 1c,e,f and 8b). In *K. pulchra*, which lacks a clavicle, the *m. cleidohumeralis* originates solely from the acromion.

The origin of the *m. scapulohumeralis superficialis* (Figures 1, 4a, 6a, 7b,c, and 8a) is continuous with the *m. scapulohumeralis profundus anterior* (Figures 1, 4b,c, 6b,c, and 8b) in most species, except for *K. pulchra* and *R. temporaria*. In the latter two species, the origin of this muscle extends proximally to the medial surface of the scapula, the paraglenoid cartilage, and the anterolateral region of the coracoid. In *Alytes obstetricans*, *R. temporaria*, and *Rhinella marina*, the *mm. scapulohumeralis superficialis* and *cleidohumeralis pars superficialis* are difficult to separate (Figure 1c,e,f).

The *m. episternohumeralis* originates neither adjacent to nor continuous with any other muscle, and is adjacent to the *m. scapulohumeralis superficialis* along its distal half in *A. obstetricans, B. orientalis, Bufo bufo, L. peronii, Rana temporaria*, and *T. resinifictrix* (Figure 8a). The origin of this muscle lies on the episternum if an episternum is present; otherwise, it arises from the anterior and ventral part of the procoracoid cartilage and the anterior and ventral part of the medial portion of the clavicle, except in *K. pulchra*, in which the clavicle is absent. In *K. pulchra*, the *m. episternohumeralis* is adjacent to but clearly distinguishable from the *m. supracoracoideus* at its origin and along its proximal half, whereas these two muscles are continuous at their distal half and at the insertion. In *Rhinella marina*, the *m. episternohumeralis* is continuous with the *m. supracoracoideus portio anterior* at the origin and along its entire length; distally, it is additionally adjacent to the *m. scapulohumeralis superficialis* at its insertion (Figure 1). In *Xenopus* spp., the *m. episternohumeralis* is well developed and, while remaining continuous at its origin on the episternum, divides distally into three portions. These are distinguished by the orientation of their fibers and by forming distinct heads, each innervated by a different nerve, that we suggest naming *portiones superficialis, intermedia*, and *profunda* (Figures 4a and 6a). The portions share a common origin, while the *portio superifialis* inserts along the posterior surface of the *crista ventralis* of the humerus, extending to its distal end; the *portio intermedia* inserts just proximal to the *crista ventralis*, on the ventral surface of the cartilaginous humerus head; and the *portio profunda* inserts on the connective tissue encapsulating the humerus head (Figure 4a). Throughout its entire length, the *m. episternohumeralis* of *Xenopus* spp. is adjacent to the anterior edge of the *m. supracoracoideus*. In *Ascaphus truei, Bombina orientalis, Bufo bufo, K. pulchra*, and *R. marina*, the *m. episternohumeralis* inserts on the distal end of the *crista ventralis* of the humerus, while it inserts proximal to the *epicondylus ulnaris* (*medialis*) in *Alytes obstetricans, L. peronii, Rana temporaria,* and *T. resinifictrix* (Figure 8a).

The *mm. cleidohumeralis*, *episternohumeralis*, and *scapulohumeralis superficialis* form a continuous muscule complex in *Ascaphus truei* and, thus, show less differentiation compared to the other species in this study. Additionally, the anteromedial part of the *m. supracoracoideus* in *A. truei* is continuous with this muscle complex, though it becomes distinct towards its insertion.

The *m. coracobrachialis* (Figures 1, 4c, 7b, and 8c,d) is divided into two units, the *pars dorsalis* and *pars ventralis*, in *A. truei*, *K. pulchra*, *R. temporaria*, and *Xenopus* spp. Both *partes* share a common insertion on the humerus. In *Xenopus* spp., the *pars dorsalis* and *pars ventralis* are clearly distinguishable at their origin but continuous at the insertion (Figures 4c and 7b), while the two *partes* are adjacent at their insertions in other species. In *Alytes obstetricans*, *Ascaphus truei*, *Bombina orientalis*, *R. temporaria*, and *Xenopus* spp., the *m. coracobrachialis* or its *pars ventralis*, if two *partes* are present and distinct, has an insertion that is nearly continuous with that of the *m. pectoralis portio coracoidea*.

The *m. subcoracoscapularis* (Figures 1, 4, 7a, and 8d) lies adjacent to the *m. coracobrachialis* in all species and is either continuous with or at least closely associated with it in *Alytes obstetricans*, *Bufo bufo*, *L. peronii* (Figure 8d), and *Rhinella marina*, as well as with the *pars dorsalis* of the *m. coracobrachialis* in *Ascaphus truei*, *K. pulchra*, and *Rana temporaria*. The *m. subcoracoscapularis* is strongly developed in *Alytes obstetricans*, *Ascaphus truei*, and *Xenopus* spp. (Figures 4 and 7a), whereas it is much thinner in *Bombina orientalis*. In these latter four species, the *m. subcoracoscapularis* inserts distally on the humerus near the *epicondylus ulnaris* (*medialis*), while in all other species, the insertion is located on the proximal half of the humerus, extending approximately from the *tuberculum mediale* to the *spina tuberculi medialis*.

The *m. pectoralis portio sternalis*, in most species, inserts directly onto the humerus and the connective tissue surrounding the tendon of the *m. coracoradialis* at the base of the *crista ventralis* of the humerus (Figures 8b,d and 1c–h). However, in *Xenopus* spp., the muscle do not insert directly onto the humerus and the connective tissue but rather through a tendon (Figure 4a). In *Rhinella marina* and *Bufo bufo*, the *m. pectoralis portio abdominalis* and *portio sternalis* shared a common tendon at their insertions. In *Xenopus* spp., the *m. pectoralis portio abdominalis* is very well developed (Figures 4a, 6a,b, and 7c). Like in all other species (Figures 1c–e,g,h and 8a,b,d), it originates from the ventral sheath of the *m. rectus abdominis*, but in this case, the origin extends posteriorly to the ventral surface of the anterior portion of the *m. sartorius*.

**Figure 9:**
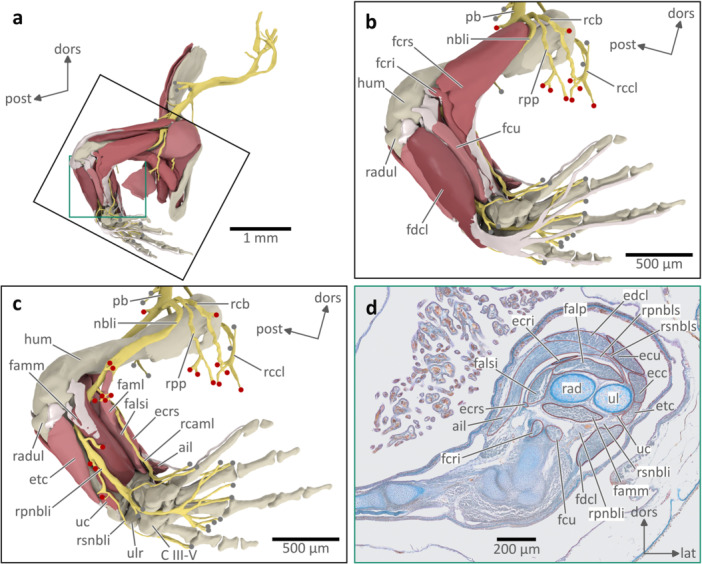
Forearm muscles with respective nerve supplies in *Limnodynastes peronii* (Gosner Stage 39, ZMH 14889). (a) Anteromedial view of left pectoral girdle and forelimb. (b, c) Anterolateral views of left forelimb. Surface derived from aligned histological serial sections, muscle layers successively removed. Spheres: nerve ending in muscle (red spheres) or cut (gray, no connection to shoulder joint muscles). Red: muscles (different shades for better visual separation of adjacent muscles); yellow: nerves; beige: skeletal element with no distinction of bone and cartilage. (d) Azan‐stained histological section of the distal part of the forearm. Red lines: muscle delineation (different shades for better visual separation of adjacent muscles). ail: m. abductor indicis longus; C: carpal; dors: dorsal; ecc: m. epicondylocubitalis; ecri: m. extensor carpi radialis carpi inferior; ecrs: m. extensor carpi radialis caput superior; ecu: m. extensor carpi ulnaris; edcl: m. extensor digitorum communis longus; etc: m. epitrochleocubitalis; falsi: m. flexor antibrachii lateralis caput inferior; falp: m. flexor antibrachii lateralis profundus; famm: m. flexor antibrachii medialis caput mediale; faml: m. flexor antibrachii medialis caput laterale; fcri: m. flexor carpi radialis caput inferior; fcrs: m. flexor carpi radialis caput superior; fcu: m. flexor carpi ulnaris; hum: humerus; nbli: n. brachialis longus inferior; nbls: n. brachialis longus superior; pb: plexus brachialis; fdcl: m. flexor digitorum communis longus; post: posterior; rad: radius; radul: radioulna; rcaml: r. cutaneus antibrachii et manus lateralis; rccl: r. coraco‐clavicularis; rcb: r. coraco‐brachialis; rpnbli: r. profundus nervi brachialis longus inferior; rpnbls: r. profundus nervi brachialis longus superior; rpp: r. pectoralis proprius; rsnbli: r. superficialis nervi brachialis longus inferior; rsnbls: r. superficialis nervi brachialis longus superior; uc: m. ulnocarpalis; ul: ulna; ulr: ulnare.

The *m. extensor digitorum communis longus* (Figures 1c,e, 4a, 5b,d, 8a, and 9d) is distally divided into three distinct heads, which we suggest naming *caput digiti medii, caput digiti annularis,* and *caput digiti minimi*, in *K. pulchra*, *Rana temporaria*, and *T. resinifictrix*. Each *caput* inserts by a tendon on the respective metacarpal: the *caput digiti medii* inserts onto the medial base of metacarpal III, the *caput digiti annularis* onto the middle of metacarpal IV, and the *caput digiti minimi* onto the lateral base of metacarpal V. In *R. temporaria* and *T. resinifictrix*, the muscular part of the *caput digiti medii* is smaller and less distinct than the other two *capita*. In *B. bufo* and *Rhinella marina* (Figure 1e), only the *capita digiti annularis* and *digiti minimi* are present and distinct, whereas no head or tendon is present that could represent the *caput digiti medii*. In these last two species, the *m. extensor digitorum communis longus* inserts only on digits IV and V. In contrast, in *L. peronii* (Figures 1a,e and 5b), the fibers of the extensor muscle are not distally divided into three heads; instead, the muscle inserts onto the metacarpals III–V by three distinct tendons. In *Alytes obstetricans, Ascaphus truei*, *Bombina orientalis*, and *Xenopus* spp. (Figure 4a), this extensor also inserts onto metacarpalia III–V, but also without forming distinct *capita*. The muscle belly remains undivided distally, and a thin fascial tendon overlays parts of the carpalia, from which three separate tendons emerge and insert individually onto the metacarpalia.

Among the carpal flexors, the *m. flexor carpi radialis* (Figures 1c,d,g, 4b,c, 5b–d, 7c, 8c,d, and 9b,d) is divided into a *caput superior* and a *caput inferior* in all examined species, except for *A. truei*, *Alytes obstetricans, K. pulchra*, and *Xenopus* spp. If two *capita* are present, the fibers of the *capita superior* and *inferior* become adjacent or even continuous distally, ultimately sharing a common tendon at their insertion. *L. peronii* (Figures 5b–d, 8c, and 9b,d) is notable for a particularly well‐developed *m. flexor carpi radialis caput superior*, which originates from the distal region of the proximal humerus head, while this muscle originates from the distal part of the humerus in the other species.

The *m. flexor antibrachii lateralis superficialis* (Figures 1c,f, 4c, 5c,d, 6d, 8c, and 9c,d) is divided into a *caput superior* and a *caput inferior* in *A. obstetricans*, *Ascaphus truei*, *B. orientalis*, *K. pulchra, L. peronii*, and *T. resinifictrix*.

The *m. flexor antibrachii medialis* (Figures 1d,h, 4c, 5d, 7c, 8e,f, and 9c,d) is divided into a *caput mediale* and a *caput laterale* in all species examined, except for *Alytes obstetricans*. In species with two capita, the *caput laterale* is markedly smaller compared to the *caput mediale* (Figures 4c and 7c).

The *m. abductor indicis longus* (Figures 1c,f, 4b, 5b,d, 8b,e, and 9c,d), of all muscles examined in this study, shows the greatest variability among species. In *Ascaphus truei* and *Alytes obstetricans*, this muscle has a triangular shape and originates from the distal half of the dorsal *sulcus intermedius* of the radioulna. It inserts via a tendon that curves around the prepollex through its medial groove and inserts along the edge of the palmar aponeurosis on the ventral side of the hand, and by minor fascial connections to the prepollex and metacarpalia II. In *Bombina orientalis*, the *m. abductor indicis longus* consists of two comparable small parts. One part originates from the radial condyle and inserts on the prepollex. The second part originates from the Y‐shaped tendon of the *m. extensor carpi radialis* and inserts with the first part on the prepollex. In *Xenopus* spp., the *m. abductor indicis longus* has no bony origin; instead, it arises from the Y‐shaped tendon of the *m. extensor carpi radialis*, then becomes adjacent to, or nearly continuous with, the *m. extensor digitorum communis longus*, and finally inserts via a tendon onto metacarpalia II (Figure 4b). In *K. pulchra, Rhinella marina* and *T. resinifictrix*, the *m. abductor indicis longus* originates from the ulna at the dorsal *sulcus intermedius* and inserts onto the metacarpalia II and the prepollex by two distinct small tendons. In *Bufo bufo, L. peronii*, and *Rana temporaria*, the *m. abductor indicis longus* is divided into a *caput superior* and a *caput inferior* at its origin (Figures 1c,f, 5b,d, 8b,e, and 9c,d). The *caput inferior* originates from the ulna at the distal half of the dorsal *sulcus intermedius* and the *caput superior* originates from the lateral epicondyle of the humerus, between the *mm. extensor carpi radialis* and *extensor digitorum communis longus*. Both *capita* unite after passing over the branching of the *n. brachialis longus superior*. The muscle then crosses laterally over the radius, passing between the *r. superficialis* and *r. profundus* of the *n. brachialis longus superior*, and inserts via a tendon onto metacarpalia II and the prepollex (Figures 5b,d and 8b).

The *m. flexor digitorum communis longus* (Figures 1b,d,g, 4a, 5d, 6a, 7a, 8a, and 9b,d) is a large and superficial muscle ventral to the radioulna. In all species, this muscle is continuous, but in early larval stages, it is possible to observe two distinct heads, which fuse during development.

### 3.2 Species‐Specific Variation in Muscle Innervation

**Figure 10:**
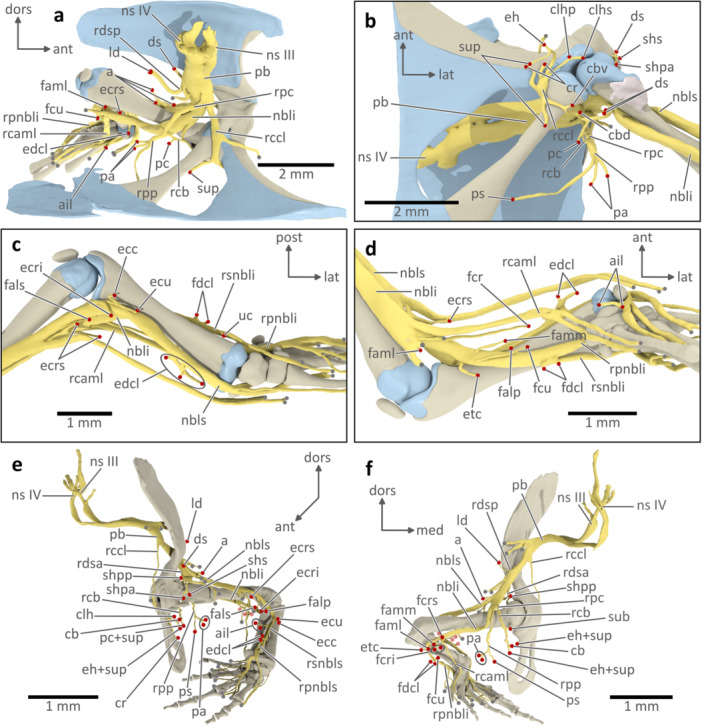
Plexus brachialis and associated nerve branches innervating the shoulder joint and forearm muscles. Spheres: nerve ending in muscle (red spheres) or cut (gray, no connection to shoulder joint or forearm muscles). Yellow: nerves. (a–d) Pattern of shoulder joint and forearm muscle innervation in *Xenopus laevis* (ZMH A05087); surfaces derived from aligned episcopic serial images; beige: bone; light blue: cartilage. (a) Medial view of left pectoral girdle half and forelimb. (b) Ventral view of the left shoulder joint. (c) Dorsal view of the left forearm. (d) Ventral view of the left forearm. (e, f) Pattern of shoulder joint and forearm muscle innervation in a *Limnodynastes peronii* larva (Gosner Stage 39, ZMH A14889); surfaces derived from aligned histological serial sections; beige: skeletal element with no distinction of bone and cartilage. (e) Anterolateral view of right pectoral girdle half and forelimb. (f) Posteromedial view of right pectoral girdle half and forelimb. a: m. anconaeus; ant: anterior; ail: m. abductor indicis longus; cb: m. coracobrachialis; cbd: m. coracobrachialis pars dorsalis; cbv: m. coracobrachialis pars ventralis; clh: m. cleidohumeralis; clhp: m. cleidohumeralis pars profunda; clhp: m. cleidohumeralis pars superficialis; cr: m. coracoradialis; dors: dorsal; ds: m. dorsalis scapulae; ecc: m. epicondylocubitalis; ecri: m. extensor carpi radialis caput inferior; ecrs: m. extensor carpi radialis caput superior; ecu: m. extensor carpi ulnaris; edcl: m. extensor digitorum communis longus; eh: m. episternohumeralis; etc: m. epitrochleocubitalis; falp: m. flexor antibrachii lateralis profundus; fals: m. flexor antibrachii lateralis superficialis; faml: m. flexor antibrachii medialis caput laterale; famm: m. flexor antibrachii medialis caput mediale; fcr: m. flexor carpi radialis; fcri: m. flexor carpi radialis caput inferior; fcrs: m. flexor carpi radialis caput superior; lat: lateral; ld: m. latissimus dorsi; med: medial; nbli: n. brachialis longus inferior; nbls: n. brachialis longus superior; ns: n. spinalis; pa: m. pectoralis portio abdominalis; pb: plexus brachialis; pc: m. pectoralis portio coracoidea; fdcl: m. flexor digitorum communis longus; ps: m. pectoralis portio sternalis; rcaml: r. cutaneus antibrachii et manus lateralis; rccl: r. coraco‐clavicularis; rcb: r. coraco‐brachialis; rdsa: r. dorsalis scapulae anterior; rdsp: r. dorsalis scapulae posterior; rpc: r. pectoralis communis; rpp: r. pectoralis proprius; rpnbli: r. profundus nervi brachialis longus inferior; rpnbls: r. profunda nervi brachialis longus superior; rsnbli: r. superficialis nervi brachialis longus inferior; rsnbls: r. superficialis nervi brachialis longus superior; shpa: m. scapulohumeralis profundus anterior; shpp: m. scapulohumeralis profundus posterior; shs: m. scapulohumeralis superficialis; sub: m. subcoracoscapularis; sup: m. supracoracoidea; uc: m. ulnocarpalis.

The muscular units of the pectoral girdle and forelimb are innervated by various branches of the brachial plexus, which is formed by spinal nerves III and IV (Figures 7c, 8, 9b,c, and 10a,b,e,f). The *r. coraco‐clavicularis* (Figures 4c, 5b,c, 7c, 8, 9b,c, and 10a,b,e,f) arises from the anterior part of this plexus, and innervates the *mm. cleidohumeralis*, *coracoradialis*, *episternohumeralis*, and *supracoracoideus* (specifically the *portio anterior* when this muscle is divided). In *Alytes obstetricans*, *Ascaphus truei*, *Bombina orientalis*, and *Xenopus* spp., a distinct branch from the *r. coraco‐clavicularis* forms an anastomosis with a branch of the *r. coraco‐brachialis* (Figures 4c and 10b). This anastomosis passes through the *m. coracobrachialis*, and in particular, through the *pars ventralis* when the *m. coracobrachialis* is subdivided, in *A. truei*, *Alytes obstetricans*, and *Xenopus* spp. In *B. orientalis* it passes through the *m. supracoracoideus*.

The next major branch of the brachial plexus, the *r. dorsalis scapulae anterior* (Figures 6d, 7c, 8c, and 10e,f) arises laterally. An anterior branch of this *ramus* innervates the *mm. dorsalis scapulae*, *scapulohumeralis superficialis*, and *scapulohumeralis profundus anterior* and *posterior*. In *Limnodynastes peronii* (Figures 8c and 10e,f) and *Trachycephalus resinifictrix*, the *m. scapulohumeralis profundus posterior* is not innervated by the *r. dorsalis scapulae anterior* but by a separate small and short nerve branch originating directly from the *plexus brachialis*. By innervating the *m. scapulohumeralis superficialis*, the *r. dorsalis scapulae anterior* innervates and passes through the *m. scapulohumeralis profundus anterior* in *A. obstetricans* and *Rana temporaria*, while it passes between these two muscles in all other species. Additionally, in all species, a posterior branch named *r. dorsalis scapulae posterior* (Figures 6d, 7c, 8f, and 10a,f) arises from the brachial plexus and innervates the *m. latissimus dorsi*.

Lateroposteriorly to the scapulohumeral joint, the brachial plexus splits into two major nerves: the *n. brachialis longus superior* (Figures 4c, 5, 8c, and 10b,d,e,f) and the *n. brachialis longus inferior* (Figures 4c, 5, 7b,c, 8f, 9b,d, and 10). The *n. brachialis longus inferior* gives rise to the *r. pectoralis communis* (Figures 7c and 10a,b,f), which distally splits into the *r. coraco‐brachialis* (Figures 5b,c, 8f, 9b,c, and 10a,b,e,f) and *r. pectoralis proprius* (Figures 4c, 5b,c, 7c, 8f, 9b,c, and 10a,b,e,f). In *A. obstetricans, Ascaphus truei*, and *B. orientalis,* the *r. pectoralis communis* innervates the *m. subcoracoscapularis*. The *r. coraco‐brachialis* innervates the *mm. coracobrachialis*, *subcoracoscapularis*, and, if present, the *m. supracoracoideus portio posterior*. In *Alytes obstetricans*, *Ascaphus truei*, and *B. orientalis*, the *r. pectoralis communis* passes between the *mm. coracobrachialis* and *subcoracoscapularis* before branching. In *Kaloula pulchra*, *L. peronii*, *R. temporaria*, *Rhinella marina*, *T. resinifictrix*, and *Xenopus* spp., only the *r. coraco‐brachialis* passes between these muscles (Figure 8e,f). The *m. pectoralis portio coracoidea* is innervated by the *r. coraco‐brachialis* in *B. orientalis*, *K. pulchra*, *L. peronii*, *Rana temporaria*, *T. resinifictrix*, and *Xenopus* spp. (Figure 10a,b,e), whereas in *Rhinella marina* it is innervated by the *r. pectoralis proprius*. In *Alytes obstetricans*, both rami (*coraco‐brachialis* and *pectoralis proprius*) contribute to the innervation of the *m. pectoralis portio coracoidea*. In the case of *Ascaphus truei*, the *m. pectoralis portio coracoidea* is not clearly distinct, making innervation assessment difficult. However, muscle fibers potentially representing the *m. pectoralis portio coracoidea* (based on their position relative to the *r. coraco‐brachialis* and *r. pectoralis proprius*) appear to be innervated by both rami, as observed in *Alytes obstetricans*.

The *r. pectoralis proprius*, the second branch of the *r. pectoralis communis*, innervates the *m. pectoralis portio abdominalis* and *portio sternalis*, and, in *A. obstetricans* and *R. marina*, also the *portio coracoidea*.

At the root of the *n. brachialis longus superior*, on the posterior aspect of the plexus brachialis, several branches emerge that innervate the various heads of the *m. anconeus* (Figure 10a,e,f). In *R. marina*, the scapular head (*caput scapulare*) is innervated by two branches directly arising from the brachial plexus. The *n. brachialis longus superior* then proceeds along the dorsal side of the forearm, innervating sequentially: *m. extensor carpi radialis caput superior*, *m. flexor antibrachii lateralis superficialis capita superior* and *inferior*, *m. extensor carpi radialis caput inferior*, *m. flexor antibrachii lateralis profundus*, *m. epicondylocubitalis*, and *m. extensor carpi ulnaris* (Figure 10c,e). The *n. brachialis longus superior* then divides at the proximal half of the radioulna into a superficial (*r. superficialis*) and a profund (*r. profundus*) branch in *A. obstetricans*, *Ascaphus truei*, *B. orientalis*, *K. pulchra*, *L. peronii*, *Rana temporaria*, *Rhinella marina*, and *T. resinifictrix* (Figure 10e). *Xenopus* spp. (Figure 10c,d) is unique in not having distinct superficial or profound antibrachial branches of the *n. brachialis longus superior*. In *Xenopus* spp., the *n. brachialis longus superior* additionally innervates the *mm. extensor digitorum communis longus* and *abductor indicis longus* (Figure 10c).

The *r. superficialis* of the *n. brachialis longus superior* (Figures 5c, 8b,c, 9d, and 10e) is located profound to the *m. extensor digitorum communis longus* and innervates this msucle in *Alytes obstetricans*, *K. pulchra*, *L. peronii*, *Rana temporaria*, *Rhinella marina*, and *T. resinifictrix*. The nerve branch then extends to the distal phalanx of digit V, running along its laterodorsal surface.

The *r. profundus* (Figures 5c, 8c, 9d, and 10e) of the *n. brachialis longus superior* lies superficial to the *m. flexor antibrachii lateralis profundus* and innervates the *mm. extensor digitorum communis longus* and *abductor indicis longus* in *Ascaphus truei* and *B. orientalis*, but only the *m. abductor indicis longus* in *Alytes obstetricans*, *K. pulchra*, *L. peronii*, *Rana temporaria*, *Rhinella marina*, and *T. resinifictrix* (Figure 10e).

The *n. brachialis longus inferior* extends on the ventral side of the forearm by running profound to the *mm. flexor carpi radialis, flexor antibrachii medialis, flexor carpi ulnaris*, and *flexor digitorum communis longus* (Figure 9c). The most proximal branch arising from the *n. brachialis longus inferior* is the *r. cutaneus antibrachii et manus lateralis* (Figures 8f, 9c, and 10a,c,d,f), which innervates the *m. flexor carpi radialis capita superior* and *inferior* in *A. obstetricans*, *Ascaphus truei*, *B. orientalis*, *K. pulchra*, *L. peronii*, *Rana temporaria*, *Rhinella marina*, and *T. resinifictrix* (Figure 10f), and additionally the *m. flexor antibrachii medialis caput laterale* in *B. orientalis*, *K. pulchra*, *Rana temporaria*, and *Rhinella marina*. In *T. resinifictrix*, the *m. flexor antibrachii medialis caput laterale* is innervated near the branching point of the *r. cutaneus antibrachii et manus lateralis*. In all species, this *ramus* is located superficial to the *m. flexor carpi radialis* (*capita superior* and *inferior*) and terminates on the lateroventral surface of the distal phalanx of digit II (Figure 9c).

From proximal to distal along the forearm, the *n. brachialis longus inferior* innervates the *m. flexor carpi radialis* in *Xenopus* spp. (Figure 10d), the *m. flexor antibrachii medialis caput laterale* in *A. truei*, *K. pulchra*, *L. peronii*, *Rana temporaria*, and *Rhinella marina* Figure 10f), the *m. flexor antibrachii medialis caput mediale* in all species except *Alytes obstetricans* and *Xenopus* spp. (Figure 10f), and the *mm. epitrochleocubitalis* and *flexor carpi ulnaris* in all species (Figure 10d,f). In *Xenopus* spp., the *m. flexor antibrachii medialis caput laterale* is innervated by a branch that originated from the *n. brachialis longus inferior* before the innervation of the *m. epitrochleocubitalis*, while the caput mediale and the *m. flexor antibrachii medialis* of *A. obstetricans* are innervated between the branches that innervate the *mm. epitrochleocubitalis* and *flexor carpi ulnaris* (Figure 10d). In *A. obstetricans*, *Ascaphus truei*, *K. pulchra*, *Rana temporaria*, *Rhinella marina*, and *T. resinifictrix*, the *n. brachialis longus inferior* also innervates the *m. flexor digitorum communis longus* proximal to branching into two terminal branches: *r. superficialis* and *r. profundus*, a branching observed across all species and located profound to the *m. flexor digitorum communis longus*.

The *r. superficialis* of the *n. brachialis longus inferior* (Figures 9c,f and 10c,d) innervates the *m. flexor digitorum communis longus* only in *Xenopus* spp. (Figure 10d). In all species, the *r. superficialis* innervates the *m. ulnocarpalis* and extends along the lateroventral external surface of the distal phalanx of digit V. In *L. peronii*, the nerve fibers innervating the *m. flexor digitorum communis longus* originate from the point where the *n. brachialis longus inferior* separated into the *r. superficialis* and *r. profundus* (Figure 9c).

The *r. profundus* of the *n. brachialis longus inferior* (Figures 8f, 9c,d, and 10a,c,d,f) innervates the *m. flexor digitorum communis longus* in *A. truei* and *B. orientalis*. In all species, this *ramus* branches in the proximal region of the ventral surface of the hand and innervates digits II–V (Figure 9c).

### 3.3 Skeletal Developmental Context

Although species‐specific variations exist, a general pattern for the development ot the skeleton can be identified (Figure 1): in the earliest stages examined herein, the pectoral girdle is composed of cartilaginous structures corresponding to the elements forming the glenoid fossa (scapula and coracoid), as well as, depending on the species, the ventral part of the suprascapula or cellular condensations preceding it. In some species, a distinct precursor of the procoracoid cartilage is also present.

As development progresses, the coracoid and procoracoid elongate ventrally and approach each other, forming the epicoracoid by the interposition of cartilaginous tissue. From the onset of metamorphic climax on, the orientation of the skeletal complex consisting of the coracoid, procoracoid, and epicoracoid gradually shifts from vertical to horizontal. Concurrently, these ventral elements rotate and extend toward their contralateral counterparts. The scapula and suprascapula, in turn, continuously expand dorsally.

The humerus, radius, and ulna develop from cartilaginous precursors. These skeletal elements elongate steadily throughout development, progressively acquiring their definitive morphology, which is notably characterized by the emergence of crests and the fusion of the radius and ulna.

The ossification of endochondral elements, as well as the formation of dermal bones such as the clavicle and cleithrum, begins at variable stages depending on the bone and species in question. In *Kaloula pulchra*, the procoracoids are reduced, and the clavicles are absent in all larval stages and adults, but there are no major effects on the rest of the skeleton (for further details, see Zhang et al. 2020). In *Bombina orientalis* and *Xenopus* spp., the episternum is not calcified (Figure 4).

Detailed descriptions of the chondrogenesis and ossification during larval development and metamorphosis are available in other works (e.g., Púgener and Maglia 1997; Maglia and Analía Púgener 1998; Baleeva 2001, 2009; Shearman 2005, 2008; Havelková and Roček 2006; Laznovsky et al. 2024) and are therefore not described nor discussed further herein.

### 3.4 Development of the Shoulder Joint and Forearm Muscles in *Rhinella Marina* and *Trachycephalus Resinifictrix*

The development of the pectoral girdle and forearm muscles is similar in *Rhinella marina* and *Trachycephalus resinifictrix* and is already in progress in the earliest larval stages considered herein (GS 32–33 for *R. marina* [ZMH A14928]; GS 33 for *T. resinifictrix* [ZMH A20006]; Figure 1a,b). In these early stages, a distinct *caput scapulare* of the *m. anconaeus* is visible laterodorsally to the shoulder joint in *R. marina*, whereas in *T. resinifictrix*, all *capita* are already separated at their origins but continuous at their insertions. The muscles dorsally spanning the shoulder joint are in both species represented by a common precursor of the *mm. dorsalis scapulae* and *latissimus dorsi*, a pre‐muscular mass corresponding to the *mm. scapulohumeralis profundus anterior* and *scapulohumeralis superficialis*, and a distinct unit representing the *m. scapulohumeralis profundus posterior*. All *scapulohumeralis* percursors are connected by a loose accumulation of undifferentiated cells. Anteroventral to the shoulder joint, a pre‐muscular mass represents the *mm. supracoracoideus* (*portiones anterior* and *posterior*), *episternohumeralis*, and *cleidohumeralis*. Posteroventrally, the *mm. subcoracoscapularis*, *coracobrachialis*, and all *portiones* of the *m. pectoralis* form a common pre‐muscle mass. The cells representing the future *m. pectoralis* appear less differentiated than those of the other muscles in the mass, such that the *m. pectoralis* can artificially be separated from the others. Located dorsally in the forearm, two proximal pre‐muscle masses are visible: the more lateral (in relation to the radius) represents the *mm. extensor carpi radialis caput superior* and *flexor antibrachii lateralis superficialis* (*capita superior* and *inferior* for *T. resinifictrix*), while the other corresponds to the *mm. extensor carpi radialis caput inferior* and *flexor antibrachii lateralis profundus*. These two pre‐muscle masses are connected by a cluster of undifferentiated cells. At the distal part of the forearm, a common precursor of the *mm. abductor indicis longus* and *extensor digitorum communis longus* is present. Lateral to the ulna, the *mm. epicondylocubitalis* and *extensor carpi ulnaris* are distinct along their entire length in *R. marina*, whereas in *T. resinifictrix*, these two muscles are continuous at their origins. Ventrally, the *mm. flexor antibrachii medialis* (*capita mediale* and *laterale*), *flexor carpi radialis* (*capita superior* and *inferior*), and *flexor carpi ulnaris* form a common pre‐muscular mass. In *R. marina*, the *m. flexor carpi ulnaris* is distinguishable from this mass at its insertion point. The *mm. epitrochleocubitalis, flexor digitorum communis longus* and *ulnocarpalis* are distinct and separate in both species.

By GS 35 in *R. marina* (ZMH A14931; Figure 1c,d), the undifferentiated cells between the *m. scapulohumeralis profundus posterior* and the *mm. scapulohumeralis superficialis* and *scapulohumeralis profundus anterior*, as well as those connecting the *mm. extensor carpi radialis caput superior* and *flexor antibrachii lateralis superficialis* (*capita superior* and *inferior*) with the *mm. extensor carpi radialis caput inferior* and *flexor antibrachii lateralis profundus*, have disappeared. The *m. latissimus dorsi* is distinct from the *m. dorsalis scapulae*, the *m. abductor indicis longus* is distinct from the *m. extensor digitorum communis longus* and the *mm. scapulohumeralis superficialis* and *scapulohumeralis profundus anterior* are separated at their insertions. The *capita scapulare* and *laterale* of the *m. anconaeus* are separated, while the *capita mediale* and *profundum* are continuous. Anteroventrally, the *m. cleidohumeralis* and the *m. supracoracoideus portio posterior* are separate from the remaining pre‐muscular mass that represents the *mm. supracoracoideus portio anterior* and *episternohumeralis*. These latter two muscles show clear separation at their origins but remain continuous at their insertions. The muscle precursor posteroventral to the shoulder joint is divided into several pre‐muscular masses: the *m. pectoralis portio abdominalis* is distinct, while the *portiones coracoidea* and *sternalis* are clearly separated at their origins, but remain semi‐continuous at their insertions, with the *m. pectoralis portio coracoidea* having a more distal insertion. The *mm. coracobrachialis* and *subcoracoscapularis* form a single muscular unit. Regarding the dorsal pre‐muscular masses of the forearm, the *mm. extensor carpi radialis caput inferior* and the *flexor antibrachii lateralis profundus*, as well as the *mm. extensor carpi radialis caput superior* and *flexor antibrachii lateralis superficialis* are, respectively, completely or almost completely separated. Ventrally, the *mm. flexor carpi radialis* (*capita superior* and *inferior*) and *flexor carpi ulnaris* are clearly separate, whereas the *m. flexor antibrachii medialis caput laterale* and the *m. flexor carpi radialis caput inferior* are not yet differentiated.

The pattern in GS 37 in *T. resinifictrix* is very similar to GS 35 in *R. marina*, yet a few differences are visible. The *m. cleidohumeralis pars superficialis* and the *m. supracoracoideus portiones anterior* and *posterior* are separated, while the *mm. episternohumeralis* and *cleidohumeralis pars profunda* remain as one pre‐muscular mass. The posteroventral muscle precursor is completely divided into the *m. pectoralis portiones abdominalis*, *coracoidea*, and *sternalis*, while the *mm. subcoracoscapularis* and *coracobrachialis* still form a common pre‐muscular mass. In the ventral part of the forearm, the *mm. flexor antibrachii medialis caput laterale*, *caput mediale*, and *flexor carpi ulnaris* are distinct, whereas the *m. flexor carpi radialis* is not yet split into two *capita*.

The specimens of *R. marina* in GS 37 and 41 (ZMH A14933; ZMH A14937; Figure 1e–h) and *T. resinifictrix* in GS 41 (ZMH A20004) show all muscular units also present in the adult specimens (Engelkes et al. 2021; Figure 1). The *mm. scapulohumeralis superficialis* and *scapulohumeralis profundus anterior* are continuous at their origin in *R. marina* and partially separated in *T. resinifictrix*, but clearly distinct at their insertions. The same pattern is observed for the *mm. episternohumeralis* and *supracoracoideus portio anterior*, as well as for the *mm. coracobrachialis* and *subcoracoscapularis*, which have distinct insertions but continuous origins in *R. marina*, and are partially separated in *T. resinifictrix*. However, in *R. marina*, these last four muscles can be artificially separated at their origin by tracing the muscle fibers from their insertions. The *m. pectoralis portiones coracoidea* and *sternalis*, as well as all *capita* of the *mm. flexor carpi radialis* and *flexor antibrachii medialis*, are completely separated. Likewise, the *m. flexor antibrachii lateralis superficialis capita superior* and *inferior* are distinct in *T. resinifictrix*. All heads of the *m. extensor digitorum communis longus* are distinct.

### 3.5 Development of the Shoulder Joint and Forearm Muscles in *Bombina Orientalis*

The development of the pectoral girdle and forearm muscles of *Bombina orientalis* is the most advanced among all species at the earliest stage considered herein (GS 32; ZMH A12427; Figures 2 and 3). Dorsal to the humerus, the *mm. dorsalis scapulae*, *latissimus dorsi,* and *capita scapulare* and *laterale* of the *m. anconaeus* are distinct. The *mm. scapulohumeralis superficialis* and *scapulohumeralis profundus anterior* are continuous at their origins but separated at their insertions. No connection is visible between these muscles and the *m. scapulohumeralis profundus posterior*. Anteroventrally, the *m. coracoradialis* is separated from all surrounding muscles, and the *m. supracoracoideus* is distinct from the pre‐muscle mass corresponding to the *mm. episternohumeralis* and *cleidohumeralis*. Posteroventrally, the precursors of the *m. pectoralis portiones abdominalis* and *sternalis* are mostly continuous but distinct at their insertions. The *portio coracoidea* is entirely distinct. The *mm. subcoracoscapularis* and *coracobrachialis* are continuous at their origins but distinct at their insertions. The *mm. extensor carpi radialis caput superior* and *flexor antibrachii lateralis superficialis* (*capita superior* and *inferior*) form one muscular precursor, as do the *mm. extensor carpi radialis caput inferior* and *flexor antibrachii lateralis profundus*. The *mm. epicondylocubitalis*, *extensor carpi ulnaris* and *extensor digitorum communis longus* are distinct. The *m. abductor indicis longus* could not be identified during muscle splitting events in *B. orientalis* due to its very small size. The *mm. flexor carpi radialis* (*capita superior* and *inferior*) and *flexor antibrachii medialis* (*capita mediale* and *laterale*) form a pre‐muscular mass that is continuous with the *m. flexor carpi ulnaris* at their origins. The *mm. epitrochleocubitalis, flexor digitorum communis longus* and *ulnocarpalis* are separate from each other.

At the next developmental stage considered in this study (GS 35; ZMH A12429; Figures 2 and 3), the *mm. episternohumeralis* and *cleidohumeralis* are distinct at their origins but almost continuous at their insertions. The *mm. coracobrachialis* and *subcoracoscapularis* are separated. The *portiones abdominalis* and *sternalis* of the *m. pectoralis* are separated. All forearm muscles are distinct and form individual muscle units.

At the latest developmental stage evaluated in *B. orientalis* (GS 41; ZMH A12435; Figures 2 and 3), the *mm. episternohumeralis* and *cleidohumeralis* are completely separated.

### 3.6 Development of the Shoulder Joint and Forearm Muscles in *Rana Temporaria*

The muscular development of the pectoral girdle and forearm in *Rana temporaria* occurs at later stages than in the other species assessed herein. At the earliest stage examined of *R. temporaria* (GS 32–33; ZMH A14736), the different clusters of pre‐muscular cells are less differentiated, resulting in fewer distinct pre‐muscular masses than in specimens of other species of comparable developmental stage. Dorsal to the humerus, a cluster of pre‐muscular cells forms the *m. anconaeus* without a distinguishable head. Proximally, the *mm. scapulohumeralis superficialis*, *profundus anterior*, *profundus posterior*, *dorsalis scapulae* and *latissimus dorsi* are represented by a single continuous pre‐muscular mass with a region of higher cell density that precedes the common tendon of the *mm. dorsalis scapulae* and *latissimus dorsi*. Ventral to the humerus, two nearly continuous pre‐muscular masses are present and separated by the distinguishable posterior portion of the future *m. coracoradialis*. The anterior mass represents the future *mm. coracoradialis*, *episternohumeralis*, *cleidohumeralis* and *supracoracoideus*, and the posterior mass the precursors of the *mm. pectoralis*, *coracobrachialis* and *subcoracoscapularis*. On the dorsal side of the forearm, two pre‐muscular masses are distinguishable. The proximal one represents the *mm. epicondylocubitalis*, *extensor carpi radialis*, *extensor carpi ulnaris*, and *flexor antibrachii lateralis superficialis* and *profundus*. This mass begins to split distally, revealing two heads. The distal pre‐muscular mass includes the *mm. abductor indicis longus* and *extensor digitorum communis longus*. One head, extending toward digit II, is distinguishable by a different muscle fiber orientation. Ventral to the radioulna, two distinct pre‐muscular masses are present. The radial mass represents the future *mm. flexor antibrachii medialis* (*capita mediale* and *laterale*), *flexor carpi radialis* and *flexor carpi ulnaris*, while the ulnar mass corresponds to the future *mm. epitrochleocubitalis* and *flexor digitorum communis longus*. At the distal end of the ulna, a cluster of pre‐muscular cells represents the *m. ulnocarpalis*, which is already separated from the *mm. epitrochleocubitalis* and *flexor digitorum communis longus* pre‐muscular mass.

In GS 34 (ZMH A14739), dorsal to the humerus, the *caput scapulare* is separate from the *m. anconaeus* at its origin but continuous at their insertions. The *mm. dorsalis scapulae* and *latissimus dorsi* form a pre‐muscular mass distinct from the *mm. scapulohumeralis* group. The *m. scapulohumeralis profundus posterior* is completely distinct, whereas the *mm. scapulohumeralis superficialis* and *profundus anterior* remain continuous. Ventral to the humerus, the *m. coracoradialis* is distinguishable but remains continuous with the common precursor of the *mm. cleidohumeralis*, *episternohumeralis* and *supracoracoideus*. Posteroventrally, the *portio abdominalis* of the *m. pectoralis* is separated at its insertion but at the origin continuous with the pre‐muscle mass representing the future *mm. pectoralis portiones coracoidea* and *sternalis*, *coracobrachialis* and *subcoracoscapularis*. The proximodorsal pre‐muscular mass of the forearm is split into a first precursor representing the *mm. extensor carpi radialis caput superior* and *flexor antibrachii lateralis superficialis*, a second precursor representing the *mm. extensor carpi radialis caput inferior* and *flexor antibrachii lateralis profundus*, and a third precursor representing the *mm. epicondylocubitalis* and *extensor carpi ulnaris*. The *mm. abductor indicis longus* and *extensor digitorum communis longus* are continuous at their origins but separated at their insertions. On the ventral side of the forearm, the *mm. epitrochleocubitalis* and *flexor digitorum communis longus* are separated from each other.

In GS 35 (ZMH A14740), the *mm. scapulohumeralis superficialis* and *profundus anterior* are starting to separate at their insertions. The *mm. dorsalis scapulae*, *latissimus dorsi,* and all *capita* of the *m. anconaeus* are separated. The *mm. episternohumeralis* and *cleidohumeralis* are separated, and the *m. supracoracoideus* remains continuous with the *m. coracoradialis*. The *portio abdominalis* of the *m. pectoralis* is distinct, whereas the *portiones coracoidea* and *sternalis* are separated at their origins but continuous at their insertions. The *m. flexor carpi ulnaris* is distinct at its insertion but remains continuous with the pre‐muscle mass representing the *mm. flexor antibrachii medialis* and *flexor carpi radialis* at its origin. The *mm. abductor indicis longus*, *epicondylocubitalis*, *extensor carpi ulnaris* and *extensor digitorum communis longus* are present as individual muscles. The *mm. extensor carpi radialis caput superior*, *inferior*, *flexor antibrachii lateralis superficialis* and *profundus* start to separate.

In GS 41 (ZMH A12870), the origin of the *m. scapulohumeralis superficialis* is separated from the *m. scapulohumeralis profundus anterior* and shifted to the medial surface of the scapula and the adjacent anterodorsal part of the coracoid. The *m. cleidohumeralis* is divided into a *pars profunda* and a *pars superficialis*, which are separated at their origins but continuous at their insertions. The *m. cleidohumeralis pars superficialis* is associated with the *mm. scapulohumeralis superficialis* and *profundus anterior* at its origin. Posteroventrally to the humerus, all *portiones* of the *m. pectoralis* are distinct. The *m. coracobrachialis* is split into a *pars ventralis* and a *pars dorsalis*, the latter being largely continuous with the *m. subcoracoscapularis*. The *mm. coracoradialis, supracoracoideus*, and all muscles of the forearm and *m. extensor digitorum communis longus* heads are distinct.

### 3.7 Development of the Shoulder Joint and Forearm Muscles in *Kaloula Pulchra*

In the earliest larval stage of *Kaloula pulchra* considered in this study (GS 31; ZMH A20007; Figures 2 and 3), the *mm. dorsalis scapulae* and *latissimus dorsi* form a distinct pre‐muscular mass that is located ventral and in close proximity to the pre‐muscle mass representing the *mm. scapulohumeralis superficialis* and *scapulohumeralis profundus anterior*. The *m. scapulohumeralis profundus posterior* is distinct and located on the dorsal surface of the humerus. The *m. anconaeus* is undivided with no visible *capita*. Ventral to the humerus, a single continuous pre‐muscular mass represents the future *mm. coracoradialis*, *episternohumeralis*, *cleidohumeralis*, *supracoracoideus*, *pectoralis*, *coracobrachialis*, and *subcoracoscapularis*. On the dorsal side of the forearm, the muscular precursor of the *mm. epicondylocubitalis* and *extensor carpi ulnaris* is distally separated from the pre‐muscle mass composed of *mm. extensor carpi radialis*, *flexor antibrachii lateralis superficialis*, and *profundus*. In this last pre‐muscle mass, the precursor of the *mm. extensor carpi radialis caput superior* and *flexor antibrachii lateralis superficialis* is distinguishable from the precursor of the *mm. extensor carpi radialis caput inferior* and *flexor antibrachii lateralis profundus*, but all muscles remain continuous. The *mm. abductor indicis longus* and *extensor digitorum communis longus* form a muscular precursor that is continuous with the muscular precursor corresponding to the *mm. epicondylocubitalis* and *extensor carpi ulnaris*. On the proximoventral side of the forearm, the future *mm. flexor antibrachii medialis* (*capita mediale* and *laterale*), *flexor carpi radialis*, and *flexor carpi ulnaris* form a continuous mass at the insertion area, but two distinct heads at the origins. The *mm. epitrochleocubitalis*, *flexor digitorum communis longus*, and *ulnocarpalis* form a discontinuous pre‐muscle mass due to the *m. ulnocarpalis,* which has already begun to separate.

In the next developmental stage considered (GS 34; ZMH A20010; Figures 2 and 3), the *m. anconaeus caput scapulare* and *caput laterale* are separated. Anteroventral to the humerus, a pre‐musclar mass represents the future *mm. episternohumeralis*, *cleidohumeralis*, *supracoracoideus*, and *coracoradialis*. Posteroventrally, a muscular precursor represents the future *mm. coracobrachialis* and *subcoracoscapularis*, and, ventral to that, a precursor corresponds to the different *portiones* of the *m. pectoralis*. The *m. epicondylocubitalis* is distinct, and the *m. extensor carpi ulnaris* is distinct at its insertion but, at its origin, remains continuous with the pre‐muscle mass representing the distinguishable precursors of the *mm. extensor carpi radialis caput superior* and *flexor antibrachii lateralis superficialis*, and the *mm. extensor carpi radialis caput inferior* and *flexor antibrachii lateralis profundus*. These precursors remain continuous distally. On the ventral side of the forearm, the *mm. epitrochleocubitalis*, *flexor digitorum communis longus*, and *ulnocarpalis* are separated.

In GS 38 (ZMH A20008; Figures 2 and 3), the *m. latissimus dorsi*, the *m. dorsalis scapulae*, and the *capita mediale* and *profundum* of the *m. anconaeus* are separated. The *mm. scapulohumeralis superficialis* and *profundus anterior* are separated at their origins but continuous at their insertions. The *mm. coracoradialis* and *cleidohumeralis* are separated, the *mm. episternohumeralis* and *supracoracoideus portio anterior* form a continuous mass, and the *m. supracoracoideus portio posterior* is separated from this mass at its origin. The *portiones abdominalis*, *coracoidea*, and *sternalis* of the *m. pectoralis* are separated, and the *mm. coracobrachialis* and *subcoracoscapularis* are mostly separated and distinguishable. Located dorsally in the forearm, the *mm. abductor indicis longus*, *extensor carpi radialis caput superior*, *caput inferior*, *extensor carpi ulnaris*, *extensor digitorum communis longus*, *flexor antibrachii lateralis superficialis*, and *profundus* are all distinct and separated. All flexor muscles on the ventral side of the forearm are separated.

At the final larval stage examined herein (GS 41; ZMH A20009; Figures 2 and 3), the *mm. scapulohumeralis superficialis* and *profundus anterior* are separated at their insertions but continuous at their origins, and the superficial muscle expanded medially onto the scapula and the adjacent anterodorsal part of the coracoid. Ventral to the humerus, the *mm. episternohumeralis* and *supracoracoideus portio anterior* are distinct at their origins but continuous at their insertions. The *m. supracoracoideus portio posterior* is distinct from the *portio anterior,* but both portions remain closely associated as the *portio anterior* covers the *portio posterior*. The *m. coracobrachialis* is divided into a *pars dorsalis* and a *pars ventralis*, with the *pars dorsalis* being continuous with the *m. subcoracoscapularis*. All *capita* of the *mm. extensor digitorum communis longus* and *flexor antibrachii lateralis superficialis* are distinct.

### 3.8 Development of the Shoulder Joint and Forearm Muscles in *Xenopus Laevis* and *Xenopus Tropicalis*

The developmental pattern of *Xenopus laevis* and *X. tropicalis* shows the greatest divergence compared to the other species examined herein. In the earliest stage examined for this genus (NF 53–54; *X. laevis*, ZMH A19320; Figures 2 and 3), dorsal to the humerus, the *m. anconaeus* is undivided, and the *mm. dorsalis scapulae* and *latissimus dorsi* form a discontinuous pre‐muscular mass, with the *m. latissimus dorsi* starting to separate at its origin. The *mm. scapulohumeralis superficialis* and *profundus anterior* are continuous, forming a single pre‐muscular mass that is continuous with the *m. scapulohumeralis profundus posterior* at their insertions. The *m. scapulohumeralis profundus posterior* is also continuous with the proximal part of the *m. anconaeus*, although an artificial separation is possible due to differences in the relative density of muscle cells. In the anteroventral region of the pectoral girdle, a condensation of pre‐muscular cells represents the future *mm. coracoradialis*, *cleidohumeralis*, *episternohumeralis*, and *supracoracoideus*. The *m. coracoradialis* is distinguishable due to a greater cell density in the region of the future tendon. Posteroventrally, two pre‐muscular masses are visible: the more ventral one represents the future *portiones abdominalis*, *coracoidea*, and *sternalis* of the *m. pectoralis*, and the more dorsal mass corresponds to the future *mm. coracobrachialis pars dorsalis* and *subcoracoscapularis*. The *m. coracobrachialis pars ventralis* is completely separated from the other pre‐muscular masses. Concerning the forearm, there are two large undivided clusters of pre‐muscular cells: one is located dorsal to the radioulna, representing all future dorsal muscles, and the second is located ventrally, representing all future ventral muscles. The dorsal pre‐muscular mass covers the entire forearm, and two heads are distinct at the insertion: the more proximal one only covers the ulna. The ventral mass shows some inhomogeneity such that the precursor of the *mm. epitrochleocubitalis*, *flexor digitorum communis longus* and *ulnocarpalis* can be identified. The precursor of these three muscles is located on the ulna, approximately parallel to the precursor of the radial‐side flexor muscles.

In the next developmental stage (NF 55; *X. laevis*, ZMH A19323; Figures 2 and 3), the *caput scapulare* of the *m. anconaeus* is distinct, and the *m. latissimus dorsi* is separated from the *m. dorsalis scapulae*. The *m. scapulohumeralis profundus posterior* remains continuous with the *m. anconaeus* at its insertion on the humerus but is separated from the *mm. scapulohumeralis superficialis* and *profundus anterior*. These two last muscles are separated at their insertions but continuous at their origins. Anteroventral to the humerus, the *mm. coracoradialis* and *supracoracoideus* are separated. Posteroventrally, the *m. pectoralis portio coracoidea* is distinct, and the *portiones abdominalis* and *sternalis* of the *m. pectoralis* form a discontinuous pre‐muscular mass with two distinct heads at the origin area. On the dorsal side of the forearm, the large pre‐muscle mass is separated into the precursor of the *mm. abductor indicis longus* and *extensor digitorum communis longus*, the precursor of the *mm. epicondylocubitalis* and *extensor carpi ulnaris*, the precursor of the *mm. extensor carpi radialis caput superior* and *flexor antibrachii lateralis superficialis*, and the precursor of the *mm. extensor carpi radialis caput inferior* and *flexor antibrachii lateralis profundus*. The *mm. epicondylocubitalis, extensor carpi ulnaris, extensor carpi radialis caput inferior* and *flexor antibrachii lateralis profundus* are separated at their insertions. On the ventral side of the radioulna, the *mm. epitrochleocubitalis, flexor digitorum communis longus* and *ulnocarpalis* are distinct, and the *m. flexor carpi ulnaris* is separated at its insertion from the pre‐muscle mass representing the *mm. flexor antibrachii medialis* (*capita mediale* and *laterale*) and *flexor carpi radialis*.

In NF 56 (*X. laevis*, ZMH A20846 *X. tropicalis*, ZMH A19277; Figures 2 and 3), all *capita* of the *m. anconaeus* are separated at their origins and continuous at their insertions. Ventral to the humerus, the *mm. cleidohumeralis* and *episternohumeralis* are distinct, and the *mm. coracobrachialis* and *subcoracoscapularis* are separated at their insertions but continuous at their origins. The *portiones abdominalis* and *sternalis* of the *m. pectoralis* are separated at their origins but continuous at their insertions. On the dorsal side of the forearm, all muscles are separated from each other except for the *mm. abductor indicis longus* and *extensor digitorum communis longus* that remain continuous at their origins.

In the final stage considered herein (NF 59; *X. tropicalis*, ZMH A19278; Figures 2 and 3), the *m. cleidohumeralis* is divided into a *pars superficialis* and a *pars profundus*, which both are continuous at their insertions. The *m. supracoracoideus* is divided into separate *portiones anterior* and *posterior*. The *portio superficialis* of the *m. episternohumeralis* is distinct, and the *portiones intermedia* and *profunda* remain continuous. Posteroventral to the pectoral girdle, the *mm. coracobrachialis pars dorsalis*, *subcoracoscapularis*, and the *portiones abdominalis* and *sternalis* of the *m. pectoralis* are distinct. Concerning the forearm, the *mm. abductor indicis longus*, *extensor digitorum communis longus*, *flexor antibrachii medialis capita mediale* and *laterale*, and *flexor carpi radialis* are separated from each other.

## 4 Discussion

### 4.1 Muscle Anatomy and Homology Hypotheses

Based on the ontogenetic development and innervation patterns described above, we suggest hypotheses of muscle homology and discuss their nomenclatural implications. Our results show that the ontogenetic muscle splitting pattern (Figures 2 and 3) is in general consistent with the embryonic muscle splitting pattern observed in tetrapods. The muscles spanning the shoulder joint are derived from two pre‐muscle masses, one located dorsal to the humerus and the other ventral, which split into individual muscles during morphogenesis (Hirasawa and Kuratani 2018; Smith‐Paredes et al. 2022 for amniotes). Similarly, dorsal and ventral pre‐muscle masses in the forearm differentiate into all antebrachial muscles (Smith‐Paredes et al. 2022). Although none of the specimens examined herein displayed only two undifferentiated pre‐muscle masses at the shoulder joint, the assessment of the splitting pattern is in accordance with the general pattern of forelimb muscle development in tetrapods: All muscle units present in late developmental stages or adult specimens are ontogenetically derived from a few pre‐muscular cell condensations and formed through subdivision of these condensations. If compared to previous work (Engelkes et al. 2021), the expanded species sample herein supports the extrapolation of this general pattern to earlier developmental stages and that the *mm. anconaeus, dorsalis scapulae, latissimus dorsi,* and the *scapulohumeralis* likely develop from a single dorsal pre‐muscle mass (Figure 2). Ventrally, the *mm. cleidohumeralis, episternohumeralis, supracoracoideus, coracoradialis, subcoracoscapularis, coracobrachialis*, and *pectoralis* possibly originate from a single pre‐muscle mass, which is supported by the observation of a single ventral pre‐muscle mass in the earliest stage of *Kaloula pulchra* considered herein (GS31; ZMH A20007; Figure 2). Similarly, only the earliest stage of *Xenopus laevis* showed two undifferentiated pre‐muscular masses in the forearm (Figure 3). Based on this observation and considering the splitting pattern of amniote forelimb muscles (Smith‐Paredes et al. 2022), it is likely that the *mm. extensor carpi radialis, extensor carpi ulnaris, extensor digitorum communis longus, abductor indicis longus, flexor antibrachii lateralis*, and *epicondylocubitalis* are derived from a single dorsal pre‐muscle mass. Likewise, a single ventral pre‐muscle mass likely gives rise to the *mm. flexor carpi radialis, flexor carpi ulnaris, flexor antibrachii medialis, epitrochleocubitalis, flexor digitorum communis longus,* and *ulnocarpalis*. This interpretation is supported by the conserved splitting pattern observed across anurans and amniotes (Smith‐Paredes et al. 2022).

The nomenclature used for the shoulder joint muscles follows the terminology proposed by Engelkes et al. (2021) and has not been modified (Table 1) as our observations using a broader species sample are consistent with the previous study. In particular, the assessment of the ontogeny of *K. pulchra*, *Trachycephalus resinifictrix*, and *Xenopus* spp., together with the addition of *Limnodynastes peronii*, further supports that the term *m. deltoideus*, as employed by Gaupp (1896), Grobbelaar (1924), and Bigalke (1927), is misleading. Our study supports the homology hypothesis that the muscular units traditionally grouped under the term *m. deltoideus*, namely the *mm. episternohumeralis*, *cleidohumeralis*, and *scapulohumeralis superficialis* (Gaupp 1896; Grobbelaar 1924; Bigalke 1927), and in some cases *scapulohumeralis profundus* (Gaupp 1896) (see Table 1), are anatomically distinct in most species and ontogenetically arise from different muscle precursors. During morphogenesis, the *mm. scapulohumeralis superficialis* and *scapulohumeralis profundus* split from the dorsal pre‐muscular mass of the shoulder joint, whereas the *mm. cleidohumeralis* and *episternohumeralis* originate from the ventral pre‐muscular mass. The different ontogenetic origins question the validity of grouping them under a single muscle name (*m. deltoideus*). Additionally, in all species examined here, the *r. dorsalis scapulae anterior* innervates the muscles of the *scapulohumeralis* complex, whereas the *mm. cleidohumeralis* and *episternohumeralis* are innervated by the *r. coraco‐clavicularis*, further supporting the abandonment of the term *deltoideus*.

Besides its historical grouping under the term *deltoideus*, the *m. episternohumeralis* has also been partially referred to as *m. mylo‐pectori‐humeralis* by Grobbelaar (1924) in *X. laevis*. In *Xenopus* spp., the *m. episternohumeralis* is well developed and differentiated into the *portiones superficialis, intermedia*, and *profunda* (Figures 4a and 6a). Grobbelaar termed the *portio superficialis* of the *m. episternohumeralis m. mylo‐pectori‐humeralis*, which he considered homologous to the *pars episternalis* of the *m. deltoideus* (Gaupp 1896) but never used this term because of the absence of an ossified episternum in *Xenopus* spp. compared to *Rana* spp. (Gaupp 1896). Grobbelaar (1924) considered the other two portions of the *m. episternohumeralis* and the *mm. cleidohumeralis* and *scapulohumeralis superficialis* to be a single muscle unit, which he referred to as *m. deltoideus*, as described above. The ontogenetic analysis of *Xenopus* spp. reveals that these three portions share a common developmental origin, arising from the ventral pre‐muscle mass. After the separation of the *m. episternohumeralis* from the *m. cleidohumeralis* (NF 56; Figure 2), the *portio superficialis* is the first to differentiate and become distinct at its origin (NF 59), whereas the *portiones intermedia* and *profunda* differentiate later in development (i.e., between NF 59 and the end of the metamorphosis). The muscle fibers of these portions are adjacent to each other and continuous at their origin but take different directions at their insertion, and are innervated by different nerves but from the same branch of the *r. coraco‐clavicularis*. These results support the homology hypotheses that these three portions are part of the *m. episternohumeralis* in *Xenopus* spp. We also observed that, in *Ascaphus truei*, *Bombina orientalis*, *Bufo bufo*, *K. pulchra*, *Rhinella marina*, and *Xenopus* spp. (*m. episternohumeralis portio superficialis*), the *m. episternohumeralis* inserts proximally on the humerus, whereas in the remaining species examined, its insertion is located more distally. These differences in the location of the insertion, which mechanically shortens the lever arm of the *m. episternohumeralis*, may reflect functional divergence among species.

Similarly, the *m. subcoracoscapularis* shows morphological disparity across species. In *B. bufo*, *K. pulchra*, *L. peronii*, *Rana temporaria*, *Rhinella marina*, and *T. resinifictrix*, this muscle is broad and short, inserting onto the proximal half of the humerus. In contrast, in *Alytes obstetricans*, *Ascaphus truei*, *Xenopus* spp., and *Bombina orientalis*, the insertion is markedly more distal, reaching as far as the *epicondylus medialis humeri* (medial epicondyle). The different locations (proximal to distal) of the insertion on the humerus likely are associated with differences in the mechanical advantage of the muscles, although the adaptive significance remains unclear and requires further investigations.

The muscle anterior to the *m. pectoralis portio abdominalis* was referred to as *m. supracoracoideus posticus* by Grobbelaar (1924), whereas the muscle deep to the *m. pectoralis portio abdominalis* was termed *m. sterno‐coracoidea* in *X. laevis*. We observed that both muscles share a tendon with the *m. pectoralis portio abdominalis* at their insertions. In addition, we found that the *m. supracoracoideus posticus* (sensu Grobbelaar 1924) and the *m. sterno‐coracoidea* both ontogenetically originate from the same posteroventral pre‐muscular mass that also gives rise to the *m. pectoralis portio abdominalis*. Based on this developmental origin, we suggest the use of the terms *m. pectoralis portio sternalis* instead of *m. supracoracoideus posticus*, and *m. pectoralis portio coracoidea* instead of *m. sterno‐coracoidea*, due to their respective origins. These revised names, already employed by Engelkes et al. (2021), highlight the close local and developmental association with the *m. pectoralis*.

Regarding the forearm, most of the muscle names used in this study follow Gaupp (1896) and are suggested to be retained with minimal modification to avoid terminological confusion. However, we identified nomenclatural inconsistencies affecting the extensor and flexor muscles. A particularly illustrative example is the *m. extensor carpi radialis*, the *capita superior* and *inferior* of which originate from two different subdivisions of the dorsal pre‐muscular mass of the forearm, yet these two muscles are still referred to with the same name (Figure 3). Our ontogenetic observations reveal an early separation of these heads: the *caput superior* arises from a pre‐muscular mass that also gives rise to the *m. flexor antibrachii lateralis superior*, whereas the *caput inferior* splits from a separate mass shared with the *m. flexor antibrachii lateralis profundus*. Despite the ontogenetic evidence indicating distinct developmental origins for the *caput superior* and the *caput inferior* of the *m. extensor carpi radialis*, we suggest retaining the widely used terminology established by Gaupp (1896). This suggestion reflects a balance between ontogenetic insight and terminological continuity, particularly in light of the close anatomical association by their Y‐shaped insertion tendon and the widespread consistent use of this nomenclature in the comparative literature. On the other hand, the use of the term *caput* can also lead to morphological ambiguity, as illustrated again by the case of the *m. extensor carpi radialis*. In *Xenopus* spp., the two muscle heads are completely separated and share neither a common origin nor a common insertion (not taking the Y‐shaped insertion tendon into account), whereas this would be expected for true muscle *capita*. However, in some species, the two heads may show a certain degree of proximal or distal fusion, which would make the use of the term *caput* more appropriate in those cases (Figure 5c).

Ritland (1955) reported for *A. truei* that no satisfactory separation could be made between the *m. flexor carpi radialis* and the *m. flexor antibrachii medialis*. By histological sections and innervation pattern, our observations confirm the presence and, despite close anatomical proximity, separation of these two muscles in all species.

A particularly illustrative example of integrating muscle morphology, ontogeny, and innervation for homology assessment is provided by the *m. abductor indicis longus*. Ritland (1955) identified a muscle unit in *A. truei* that he considered homologous to the *m. abductor indicis longus* (sensu Gaupp 1896) in *Rana* spp., which Ritland referred to as *m. supinator manus* based on its presumed supinating function. Given the anatomical variation of the *m. abductor indicis longus* observed among the species examined here, as well as the observed innervation, we suggest using the term *m. abductor indicis longus* for this muscle in *A. truei* to reflect the likely homology with the corresponding muscle in the other species considered herein.

The interspecific anatomical variation observed in this study for the *m. adbuctor indicis longus* is one of the most pronounced compared to the variation observed for other muscles. The muscle shows considerable variation in its associations with the prepollex, metacarpal II, and adjacent structures, as well as in the degree of subdivision observed among species, with some species exhibiting distinct *capita superior* and *inferior*. Despite the morphological differences regarding the locations of origins and insertions, the suggested homology and distinction of this muscle with the *m. extensor digitorum communis longus* across species are well supported by the ontogenetic observations (as suggested by Manzano et al. 2008). This morphological disparity may reflect functional adaptations associated with the progressive medial rotation of the anuran hand (Ritland 1955; Sigurdsen et al. 2012; Fabrezi et al. 2017), but also may be the result of selective pressures related to amplexus, given the central role of digit II and the prepollex in nuptial grasping (Emerson 1991; Lee Clark and Peters 2006; Manzano et al. 2008). Further investigations integrating functional, behavioral, and possibly sexually dimorphism of this muscle in combination with an extended species sample are needed to shed light on the evolutionary drivers underlying its remarkable variability.

The *m. extensor digitorum communis longus* represents another notable example in which interspecific morphological variation, combined with ontogeny, supports revised homology hypotheses and nomenclature. In *Alytes obstetricans, Ascaphus truei, B. orientalis*, and *Xenopus* spp., the muscle is a single continuous muscular unit that extends distally with a broad, thin tendon, which distally splits into three tendinous heads inserting onto the metacarpals of digits III–V (Figure 7a). In *K. pulchra, Rana temporaria*, and *T. resinifictrix*, the *m. extensor digitorum communis longus* is distally divided into two or three distinct muscle heads inserting onto the digits III–V. As these muscle heads are distinct at their insertion and their homology is supported by ontogenetic observations, we suggest naming them, from digit III–V, *caput digiti medii*, *caput digiti annularis*, and *caput digiti minimi*. Among the species examined, particularly distinctive conditions were observed in *Bufo bufo* and *R. marina*, in which the muscle is distally divided into two muscle heads, the *caput digiti medii* and its tendon being absent. In *L. peronii*, by contrast, the muscle is distally not subdivided into distinct *capita*, whereas the insertion tendons of the metacarpals III–V are completely separated and distinct (Figure 5b). This diversity may reflect functional adaptations associated with the progressive medial rotation of the anuran's hand (Ritland 1955; Sigurdsen et al. 2012; Fabrezi et al. 2017), possibly enabling finer directional control of digital extension (Hu et al. 2015). Alternatively, this diversity across species may be the result of selection pressures related to species ecology or behavior (e.g., amplexus).

### 4.2 Limitation

Our species sampling was limited and reflected a compromise between including representatives of major anuran clades across the phylogeny and the workload associated with the chosen methods. This limitation also affected our sampling of developmental stages, as only a single specimen per species represented each of the considered stages. Thus, the anatomical differences observed within species may not necessarily reflect clade‐level characteristics but rather intraspecific variation. Likewise, differences in the timing of developmental events (e.g., muscle splitting pattern) may result from intraspecific variation rather than true interspecific differences. Nevertheless, interspecific variation in the timing of pectoral girdle and muscle development has already been reported in other anuran species (Baleeva 2001; Soliz et al. 2018), and our anatomical and developmental observations were consistent within each species.

The innervation patterns used for the identification and assessment of homology between muscle units were described using two specimens per species whenever possible, but only based on a single specimen in some species (Supporting Information S1: Material S1). The variations observed across species may reflect intraspecific differences rather than species‐specific traits, as previously reported in other anuran taxa (Manzano et al. 2017).

With one specimen per developmental stage, sexual dimorphism was not considered in our study and may have contributed to within‐ and between‐species variation in our results. Sexual dimorphism has already been reported in anurans for the humerus (Lee 2001; Padhye et al. 2015; Petrović et al. 2017), as well as for specific muscles of the pectoral girdle (Oka et al. 1984; Emerson 1990; Lee 2001), the hand (Emerson 1991; Lee Clark and Peters 2006; Manzano et al. 2008), and forearm muscles (Gaupp 1896; Ritland 1955; Mao et al. 2014), highlighting the need for further investigation.

Despite these limitations, we are confident that our sample is sufficient to identify the distinct muscular units of the shoulder joint and forearm and to evaluate their interspecific homologies based on ontogenetic patterns and innervation. This confidence is supported by the consistency of our observations across developmental stages within each species and among closely related species, which allows to reconstruct the ontogenetic splitting patterns of the pre‐muscular masses. We believe that including additional specimens would provide limited further insight into the ontogenetic development of these muscles. However, increasing the number of adult specimens and expanding the species sample could substantially improve our understanding of the interspecific muscle variation by allowing to better assess intraspecific variation and reconstruct evolutionary pathways. In contrast, including earlier developmental stages seems unnecessary, as the specimens already analyzed were sufficient to identify homologous pre‐muscular masses. In some species, as for example observed in *Xenopus* spp., the number of pre‐muscle cells in the forearm may even be too low to allow for meaningful identification of pre‐muscular organization in early developmental stages.

## 5 Conclusion

Our study demonstrates that the pre‐muscular masses giving rise to the shoulder joint and forearm musculature in anurans follow a rather conserved splitting pattern, as it has also been observed in other tetrapods (Smith‐Paredes et al. 2022). This ontogenetic pattern, together with the innervation pattern, allowed us to suggest well‐supported homology hypotheses and resolve the inconsistencies persistent in the literature by applying homology criteria to establish a consistent nomenclature. This study extends the work of Engelkes et al. (2021) by incorporating additional species across the anuran phylogeny as well as the forearm musculature. Using the same name for muscles in different species implies homology, which, in some previous works, is not given or not well‐supported, which is why the terminology we suggest is based on a detailed assessment of ontogenetic development and innervation. The nomenclature initially proposed by Engelkes et al. (2021) is here consolidated and expanded by the forearm muscles, while mostly maintaining established terms to minimize changes and avoid impeding the understanding of forelimb muscle evolution.

This study highlights the importance of critically reviewing published anatomical descriptions before using them for comparative purposes in new studies. For example, the different ontogenetic origins of the muscle units traditionally considered as portions of the *m. deltoideus* complicate their homologization with a single muscle. Moreover, most muscles are designated by terms that describe their presumed function, but this function may vary between species, making the homologization across species more difficult and the muscle names less appropriate and thereby potentially confusing in some species. The most striking example is the *m. abductor indicis longus*, for which the homologization without developmental assessment is difficult due to its large morphological diversity and varying functions, ranging from a presumed abductor of the index, to an extensor (Manzano et al. 2008), or even a supinator of the hand (Ritland 1955). We believe that the assumptions of homology must be rigorously evaluated before using it as a base to formulate or support evolutionary hypotheses, as only well‐supported homologies between muscle units across taxa allow for a reliable reconstruction of ancestral character states. Considerable morphological variations were observed in some muscles of the shoulder joint and forearm. The reconstruction of muscle evolution and, subsequently, the assessment of the biomechanical implications and adaptive significance of morphological variations remain for future studies with a more comprehensive species sample.

## Author Contributions

R.S.M. conceived the study, performed most of the histological serial sectioning and digitization, conducted the segmentation and 3D reconstructions, generated the 3D models and figures, analyzed the data, and drafted the manuscript. K.E. provided additional histological and episcopic data. R.S.M. and K.E. discussed the results. K.E. provided resources and acquired funding. Both authors critically revised the manuscript and approved the final version.

## Conflicts of Interest

The authors declare no conflicts of interest.

## Supporting information


Supporting File

## Data Availability

The data that support the findings of this study are available in the Supporting Information S1: material of this article.
