# Supplementary material for: Ontogeny and Homology of the Shoulder Joint and Forelimb Muscles in Frogs and Toads (Lissamphibia: Anura)
Source: J Morphol. 2026 Aug 19;287(8):e70158. doi: 10.1002/jmor.70158 (PMC13487509; doi:10.1002/jmor.70158)
Supplement: Supplementary file 1 [file JMOR-287-e70158-s001.docx]

**Supplementary material S1** Specimens, developmental stage, and methods. Staging after Gosner (1960) (GS), and Nieuwkoop and Faber (1994) (NF). CAS: California Academy of Sciences; ZMH: Zoologisches Museum Hamburg

| Specimen (collection number | Developmental stage | Method | Remark |
| --- | --- | --- | --- |
| *Alytes obstetricans* (ZMH A12442) | Subadult/adult | Episcopic microtomy | dataset from Engelkes *et al.* (2018) |
| *Ascaphus truei* (CAS 252678) | Adult | µCT lead stained | <https://media.leibniz-lib.de/search/#/detail/343494> |
| *Ascaphus truei* (ZMH A09807) | GS 42 | Histology | <https://doi.org/10.25592/uhhfdm.1754> |
| *Bombina orientalis* (ZMH A12427) | GS 32 | Histology | <https://doi.org/10.25592/uhhfdm.1768> |
| *Bombina orientalis* (ZMH A12429) | GS 35 | Histology | <https://doi.org/10.25592/uhhfdm.1770> |
| *Bombina orientalis* (ZMH A12435) | GS 41 | Histology | <https://doi.org/10.25592/uhhfdm.1772> |
| *Bufo bufo* (ZMH A04660) | Adult | µCT iodine stained | <https://doi.org/10.25592/uhhfdm.1788> |
| *Kaloula pulchra* (ZMH A20007) | GS 31 | Histology | <https://media.leibniz-lib.de/search/#/detail/343488> |
| *Kaloula pulchra* (ZMH A20010) | GS 34 | Histology | <https://media.leibniz-lib.de/search/#/detail/343489> |
| *Kaloula pulchra* (ZMH A20008) | GS 38 | Histology | <https://media.leibniz-lib.de/search/#/detail/343493> |
| *Kaloula pulchra* (ZMH A20009) | GS 41 | Histology | <https://media.leibniz-lib.de/search/#/detail/343497> |
| *Kaloula pulchra* (ZMH A15064) | Subadult/adult | Episcopic microtomy | <https://media.leibniz-lib.de/search/#/detail/343428> |
| *Limnodynastes peronii* (ZMH A14888) | GS 36 | Histology | <https://media.leibniz-lib.de/search/#/detail/343430> |
| *Limnodynastes peronii* (ZMH A14889) | GS 39 | Histology, 3D reconstruction | <https://media.leibniz-lib.de/search/#/detail/343504> |
| *Limnodynastes peronii* (ZMH A19233) | GS 43 | Histology | <https://media.leibniz-lib.de/search/#/detail/343511> |
| *Rana temporaria* (ZMH A14736) | GS 32-33 | Histology | <https://doi.org/10.25592/uhhfdm.1824> |
| *Rana temporaria* (ZMH A14739) | GS 34 | Histology | <https://doi.org/10.25592/uhhfdm.1826> |
| *Rana temporaria* (ZMH A14740) | GS 35 | Histology | <https://doi.org/10.25592/uhhfdm.1828> |
| *Rana temporaria* (ZMH A12870) | GS 41 | Histology | <https://doi.org/10.25592/uhhfdm.1822> |
| *Rana temporaria* (ZMH A11310) | Adult | µCT iodine stained | <https://media.leibniz-lib.de/search/#/detail/343432> |
| *Rhinella marina* (ZMH A14928) | GS 32-33 | Histology, 3D reconstruction | <https://doi.org/10.25592/uhhfdm.1830> |
| *Rhinella marina* (ZMH A14931) | GS 35 | Histology, 3D reconstruction | <https://media.leibniz-lib.de/search/#/detail/343433> |
| *Rhinella marina* (ZMH A14933) | GS 37 | Histology, 3D reconstruction | <https://doi.org/10.25592/uhhfdm.1834> |
| *Rhinella marina* (ZMH A14937) | GS 41 | Histology | <https://doi.org/10.25592/uhhfdm.1836> |
| *Trachycephalus resinifictrix* (ZMH A20006) | GS 33-34 | Histology | <https://media.leibniz-lib.de/search/#/detail/343490> |
| *Trachycephalus resinifictrix* (ZMH A20002) | GS 37 | Histology | <https://media.leibniz-lib.de/search/#/detail/343491> |
| *Trachycephalus resinifictrix* (ZMH A20004) | GS 41 | Histology | <https://media.leibniz-lib.de/search/#/detail/343514> |
| *Xenopus laevis* (ZMH A19320) | NF 53-54 | Histology | <https://media.leibniz-lib.de/search/#/detail/343441> |
| *Xenopus laevis* (ZMH A19323) | NF 55 | Histology | <https://media.leibniz-lib.de/search/#/detail/343492> |
| *Xenopus laevis* (ZMH A20846) | NF 56 | Histology | <https://media.leibniz-lib.de/search/#/detail/343498> |
| *Xenopus laevis* (ZMH A05087) | Subadult/adult | Episcopic microtomy, 3D reconstruction | <https://media.leibniz-lib.de/search/#/detail/343534> |
| *Xenopus tropicalis* (ZMH A19276) | NF 54 | Histology | <https://media.leibniz-lib.de/search/#/detail/343500> |
| *Xenopus tropicalis* (ZMH A19277) | NF 56 | Histology | <https://media.leibniz-lib.de/search/#/detail/343476> |
| *Xenopus tropicalis* (ZMH A19278) | NF 59 | Histology, 3D reconstruction | <https://media.leibniz-lib.de/search/#/detail/343539> |
